# Cloud-assisted IoRT framework for latency-aware analytical kinematic control of a six-DOF robotic manipulator

**DOI:** 10.1038/s41598-026-65330-w

**Published:** 2026-08-14

**Authors:** Mohamed S. Elhadidy, Sarah M. Ayyad, Waleed Shaaban, Waleed S. Abdalla, Mahmoud M. Saafan

**Affiliations:** 1 Mechatronics Engineering Department, Faculty of Engineering, Mansoura University, Mansoura, 35516 Egypt; 2 Department of Mechatronics Engineering, Faculty of Engineering, Horus University, New Damietta, 34517 Egypt; 3 Computers and Control Systems Engineering Department, Faculty of Engineering, Mansoura University, Mansoura, 35516 Egypt; 4 Faculty of Engineering, Mansoura National University, Gamasa, 35712 Egypt; 5 Mechanical Power Engineering Department, Faculty of Engineering, Mansoura University, Mansoura, 35516 Egypt; 6 Department of Mechanical Design and Production Engineering, Faculty of Engineering, Zagazig University, Zagazig, 44511 Egypt

**Keywords:** Internet of Robotic Things, Cloud robotics, Six-DOF manipulator, Cloud–edge control, Industrial robotics, Deterministic robotic control, Engineering, Mathematics and computing

## Abstract

The Internet of Robotic Things (IoRT) has introduced new possibilities for distributed and scalable robotic control by combining industrial manipulators with cloud-based computation and networked communication infrastructures. However, the deployment of cloud-assisted robotic systems requires accurate kinematic modeling, reliable communication, and latency-aware execution, particularly when kinematic computations are distributed between cloud and edge layers. This study presents an IoRT-based cloud-assisted control framework for a six-degree-of-freedom industrial robotic manipulator, using the PUMA 560 as a benchmark platform. The framework adopts the established analytical forward- and inverse-kinematics formulations of the manipulator as a non-iterative and interpretable computational core rather than proposing a new kinematic formulation. The principal contribution lies in integrating these analytical models into a distributed cloud–edge architecture in which inverse-kinematics computation, evaluation of multiple solution branches, and trajectory generation are performed in the cloud, while command validation, safety monitoring, and motion execution remain at the edge. Candidate inverse-kinematics branches are evaluated according to joint-limit feasibility, proximity to singular configurations, and joint-space continuity. In the proposed architecture, computationally intensive kinematic calculations are performed in the cloud, while motion validation, safety monitoring, and command execution remain at the edge level to ensure operational reliability. MATLAB-based simulations are conducted to assess forward–inverse–forward model consistency, joint-space trajectory continuity, and communication timing under the adopted cloud–edge model. The kinematic consistency evaluation yields root-mean-square position and orientation reconstruction errors of 0.066 mm and 0.206°, respectively, over the representative test poses. These values quantify the internal agreement between the implemented analytical inverse- and forward-kinematics models under the adopted numerical settings and should not be interpreted as physical robot tracking accuracy. Furthermore, the multi-branch inverse-kinematics selection strategy reduces total joint-space motion by approximately 54.3% relative to random branch selection, improving trajectory smoothness and potentially reducing unnecessary joint actuation. The communication analysis shows that the hybrid architecture achieves an average modeled delay of 0.210 s with no timeout events during the evaluated sequences. These findings demonstrate the simulation-level feasibility of integrating analytical kinematic control into a cloud-assisted IoRT architecture while maintaining numerical consistency, effective solution selection, and predictable execution under the evaluated communication conditions.

## Introduction

Robotics is an interdisciplinary domain concerned with the design and deployment of machines that integrate mechanical structures, sensing, computation, and control to perform tasks autonomously or semi-autonomously. The field has evolved from early industrial automation toward intelligent robotic systems capable of perception, decision-making, and adaptive interaction with their environments ^1^. This evolution has been driven by advances in electronics, computing, and artificial intelligence, enabling robots to operate effectively in both structured and unstructured settings ^2^. Robots are commonly defined as programmable electromechanical systems that combine sensing, computation, and actuation to execute physical tasks with varying levels of autonomy, distinguishing them from conventional fixed automation through their reconfigurability and decision-making capabilities ^3^.

**Fig. 1:**
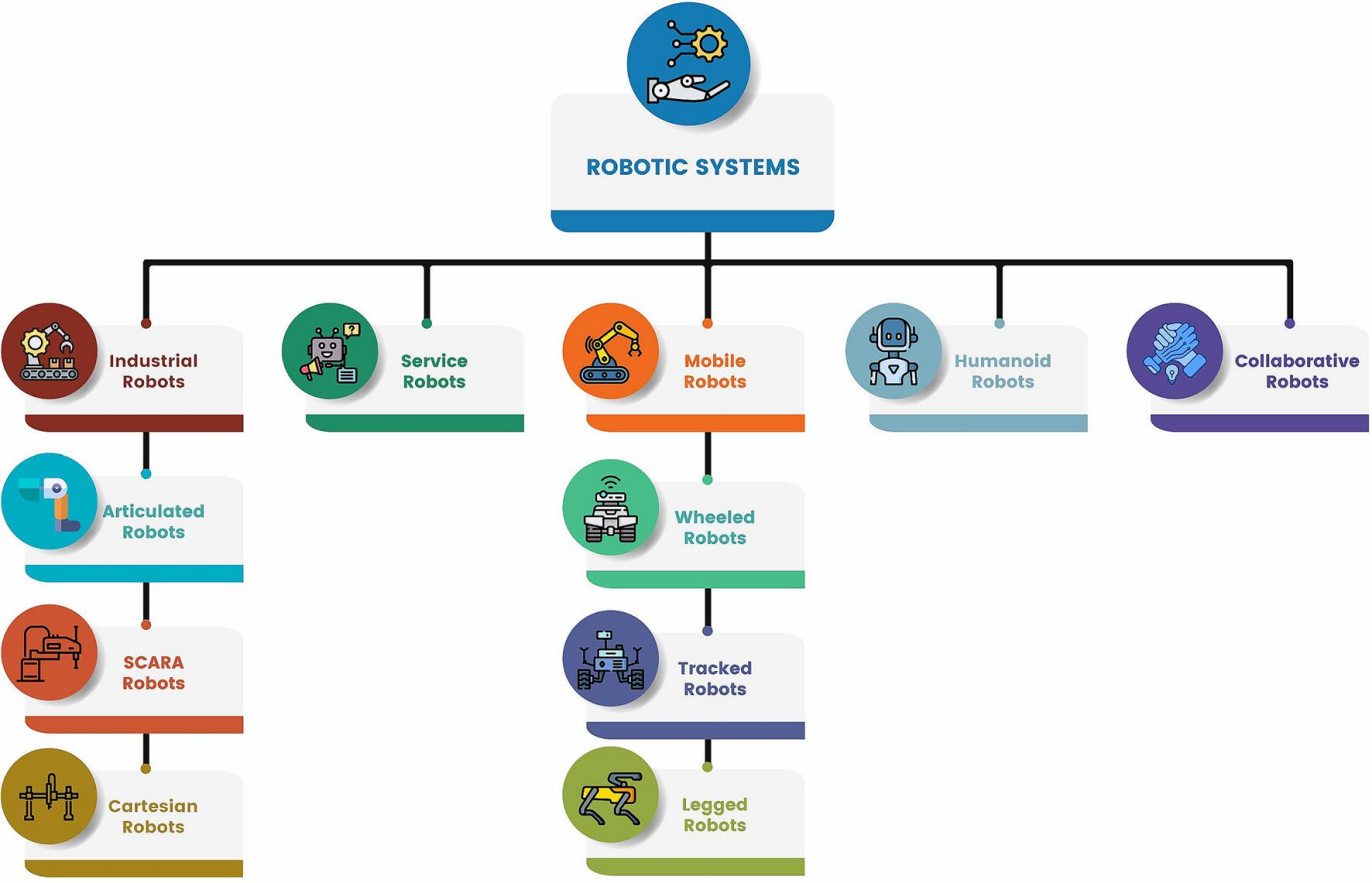
Classification of Robotic Systems.

Robotic systems are commonly classified into industrial, service, mobile, humanoid, and collaborative robots based on their mechanical structure, functional roles, and interaction characteristics ^4^. This classification reflects the diversity of robotic platforms and application requirements, ranging from high-precision industrial automation to human-centered interaction and safe human–robot collaboration. The increasing variety of robotic structures and operating environments underscores the need for adaptable modeling and control strategies capable of accommodating different kinematic configurations and autonomy levels, as illustrated in Fig. 1^5^.

**Fig. 2:**
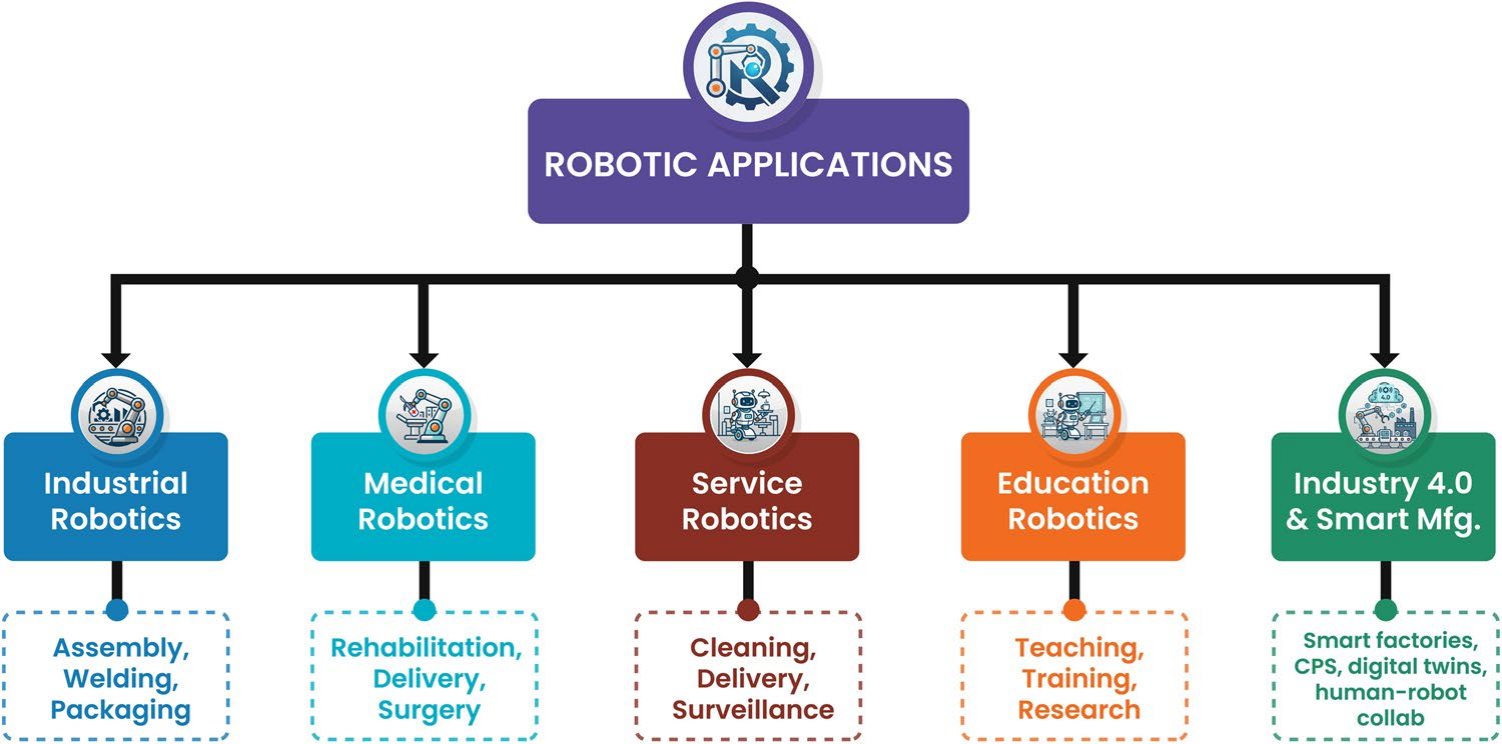
Major application domains of robotic systems.

In practical applications, robots are widely deployed across industrial, medical, service, educational, and public domains. Industrial robots perform tasks such as assembly, welding, painting, and material handling, supporting high repeatability and flexible smart manufacturing systems aligned with Industry 4.0 paradigms ^6^. In the medical field, robotic systems are used in surgery, rehabilitation, diagnostics, and telemedicine, enabling enhanced precision and minimally invasive procedures. Service and educational robots further extend robotic deployment to domestic assistance, commercial operations, public safety, and hands-on learning environments ^7^. Figure 2 illustrates the major application domains of robotic systems ^8^.

A robot arm, or robotic manipulator, is a serial kinematic mechanism composed of rigid links, joints, actuators, sensors, and a control unit that collectively enable precise positioning and orientation of an end effector within a three-dimensional workspace ^9^. Its motion capability is defined by the number of degrees of freedom and joint types, typically revolute or prismatic, which directly influence dexterity, reachability, and manipulability. As the number of degrees of freedom increases, kinematic modeling and control complexity also increase, making kinematic analysis a fundamental aspect of manipulator design and operation ^10^. Among industrial serial manipulators, the PUMA 560 remains a widely adopted benchmark platform due to its anthropomorphic structure and spherical wrist configuration, which facilitate analytical forward and inverse kinematics modeling and make it particularly suitable for evaluating deterministic kinematic and control strategies ^11^.

One of the most common robotic arm applications is the industrial serial manipulator, which is capable of full spatial pose control through its articulated structure. The decoupled kinematic design of such manipulators enables closed-form analytical inverse kinematics solutions, making them suitable for research, education, and complex industrial tasks requiring high dexterity and manipulability ^12^. Robot arm control focuses on generating actuator commands for accurate motion execution using position, velocity, and torque control schemes. The nonlinear behavior of multi-degree-of-freedom manipulators, combined with modeling uncertainties and networked control constraints, necessitates precise kinematic modeling and robust control strategies to ensure stability and performance ^13^.

Kinematic analysis establishes the mathematical relationship between joint space and task space and forms the foundation for robotic motion planning and control. Forward kinematics (FK) determines the position and orientation of the end effector from known joint variables using geometric modeling, commonly based on the Denavit Hartenberg formulation of homogeneous transformation matrices. Due to its deterministic and computationally efficient nature, FK is well suited for real-time control, calibration, workspace analysis, and validation of inverse kinematics solutions ^14^. Inverse kinematics computes the joint variables required to achieve a desired end-effector pose and is inherently more complex due to nonlinear and coupled equations. While analytical solutions provide deterministic computation, numerical and optimization-based methods offer greater flexibility at the cost of increased computational demand and potential convergence issues, particularly in the presence of joint limits and kinematic singularities ^15^.

The Internet of Robotic Things (IoRT) extends the concept of the Internet of Things by integrating robotic systems with networked sensing, communication, and cloud-based computation, enabling robots to operate as intelligent, interconnected agents within distributed cyber–physical environments ^16^. In an IoRT architecture, robotic platforms are typically organized across multiple layers, including the physical layer (robot manipulators, sensors, and actuators), the edge layer (local controllers and edge processors for real-time execution), and the cloud layer (remote servers for data storage, high-level computation, coordination, and decision-making). This layered architecture facilitates scalable deployment, collaborative operation, and efficient resource utilization by offloading computationally intensive tasks while maintaining low-latency local control ^17^. Within such architectures, forward and inverse kinematics play a critical role by providing the mathematical interface between high-level task planning executed in the cloud and low-level joint-space command execution performed at the robot or edge level. Deterministic analytical kinematic models are particularly well suited for IoRT-based control, as they enable compact command transmission, reliable pose reconstruction, and predictable execution under network delays and bandwidth constraints. These properties are essential for ensuring stability, safety, and real-time responsiveness in distributed robotic systems ^18^.

Despite significant advancements in robotic manipulators, kinematic modeling, and cloud-enabled control systems, several limitations remain in the existing literature that hinder the development of reliable and scalable IoRT-based robotic solutions:

- Most existing approaches rely heavily on numerical or data-driven inverse kinematics methods, which may suffer from convergence issues, high computational cost, and limited interpretability, particularly in real-time and networked control environments.
- There is a noticeable lack of analytical forward and inverse kinematics formulations integrated within cloud-based or IoRT-enabled robotic control frameworks, especially for manipulators with limited degrees of freedom.
- Current IoRT and cloud robotics studies often emphasize high-level planning, monitoring, or perception, while low-level kinematic computation and execution are still predominantly performed locally on embedded controllers.
- While the PUMA 560 is a well-studied 6-DOF industrial robot, many recent works favor numerical or learning-based inverse kinematics methods, leading to underutilization of its analytical closed-form kinematic properties.

It should be emphasized that the analytical forward- and inverse-kinematics formulations of the PUMA 560 are classical and well established in the robotics literature. Accordingly, the novelty of this study does not reside in re-deriving these kinematic equations. The equations are presented to establish a complete, internally consistent, and reproducible mathematical basis for their implementation within the proposed system. The principal contribution is instead the system-level integration of established, non-iterative analytical kinematics into a cloud–edge IoRT control architecture, including explicit cloud–edge task allocation, evaluation and selection of multiple inverse-kinematics branches, local constraint and safety validation, mathematical decomposition of the control-loop delay, and comparative analysis of alternative communication configurations under a common simulation environment.

The main contributions of this paper can be summarized as follows:

- Implementation and simulation-based verification of the established analytical forward- and inverse-kinematics models of the PUMA 560 as a non-iterative computational module suitable for cloud-assisted execution, including the generation and evaluation of multiple inverse-kinematics solution branches.
- Development of a layered cloud–edge IoRT framework that assigns analytical kinematic computation, solution selection, and trajectory generation to the cloud while retaining joint-limit verification, safety checks, emergency handling, and command execution at the edge.
- Formulation of a cloud–edge timing model that separates uplink communication delay, cloud-based inverse-kinematics computation time, downlink communication delay, and local execution time, thereby enabling a structured assessment of the temporal behavior of the proposed architecture.
- Comparative simulation-based evaluation of MQTT-only, REST-only, and hybrid MQTT–REST communication configurations under identical kinematic and execution conditions, showing that the hybrid configuration achieves an average control-loop delay of 0.210 s with lower variability than the alternative configurations.
- Comprehensive performance evaluation demonstrating that multi-branch inverse-kinematics solution selection reduces total joint-space motion by approximately 54.3% relative to random branch selection, while the hybrid cloud–edge communication architecture achieves an average delay of 0.210 s under the evaluated conditions.

The remainder of this paper is organized as follows. Section "Introduction" introduces the study and reviews related work. Section "Material and Methods" presents the materials and methods, which include the kinematic analysis of the robotic manipulator using mathematical modeling, forward and inverse kinematics, and the proposed IoRT-based robotic control framework. Section "Edge-side execution and validation" presents the results and discussion. Finally, Section "Feedback and closed-loop operation" concludes the paper and outlines future work.

### Literature review

This section reviews studies directly related to analytical forward and inverse kinematics, multi-branch inverse-kinematics solution methods, optimization- and learning-based alternatives, and IoRT-enabled robotic control. The comparison focuses on computational determinism, constraint handling, interpretability, communication integration, and suitability for cloud–edge execution. Particular attention is given to the limited integration of closed-form analytical kinematics with networked robotic architectures, which constitutes the principal research gap addressed in this study.

Guo et al. ^19^ developed a closed-form inverse kinematics solution for a redundant 7-DOF manipulator using DH-based modeling and virtual arm angle decoupling. The method avoids Jacobian computations and shows computational efficiency, but it was validated only in simulation and lacks analysis under dynamic conditions or across different robot architectures. Baranov et al. ^20^ presented an analytical forward kinematics formulation for a six-axis industrial manipulator based on classical DH parameters. The work demonstrates high positional accuracy and educational value, yet it is limited to direct kinematics and does not address inverse kinematics or real-time adaptive control. Karhan and Bingul ^21^ proposed an automated technique for extracting Denavit–Hartenberg parameters directly from robot geometry. Their approach reduces manual modeling errors and improves usability, but it focuses solely on kinematic parameterization without integration into control or distributed robotic frameworks. Rettig et al. ^22^ reformulated robotic kinematics using conformal geometric algebra as an alternative to matrix-based methods. While mathematically compact and expressive, the approach remains theoretical and does not address control implementation or IoRT-based deployment.

Wang et al. ^23^ introduced a multi-objective genetic algorithm for inverse kinematics and trajectory optimization of a 6-DOF robot. Although improved trajectory quality was achieved, the method suffers from high computational cost and parameter sensitivity, limiting real-time applicability. Li and Luo ^24^ proposed a hybrid inverse kinematics and motion planning framework combining gradient projection with RRT*. The method enhances feasibility in constrained environments, but iterative optimization increases computation time and dependence on initial conditions. Shen and Wang ^25^ employed a genetic algorithm integrated with kinematics modeling and analytic hierarchy processes to improve trajectory smoothness and joint-limit compliance. Despite improved path quality, the approach relies heavily on heuristic design and lacks deterministic guarantees. More recent works have explored hybrid evolutionary strategies to further enhance optimization performance. Ting Wang et al. ^26^ proposed an improved differential evolution algorithm combined with whale optimization for inverse kinematics of an ABB industrial robot, achieving higher convergence accuracy compared to standalone metaheuristics. Elhadidy et al. ^27^ similarly investigated particle swarm optimization and hybrid solvers for multi-DOF manipulators, demonstrating robustness under complex constraints at the expense of increased computational cost and limited interpretability of the resulting joint solutions.

Ibrahim and Ali ^28^ proposed a deep neural network–based inverse kinematics model that learns joint configurations from task-space inputs. The approach enables fast inference, but its performance depends strongly on training data quality and lacks proven generalization and constraint guarantees. Calzada-Garcia et al. ^29^ conducted a comprehensive review of deep learning–based inverse kinematics methods, highlighting their potential for high-DOF manipulators. The study identifies key limitations including poor interpretability, dataset bias, and the absence of formal feasibility guarantees. Cuadros Zegarra et al. ^30^ presented an IoRT middleware framework to enable interoperability among heterogeneous robotic systems. While improved communication and modularity were demonstrated, real-time performance, latency effects, and dynamic adaptability were not thoroughly evaluated. Arefin et al. ^31^ proposed an early cloud-based IoRT robotic arm control system enabling remote monitoring and command execution. The framework demonstrates basic cloud–robot connectivity but lacks analytical kinematics integration and analysis of communication delays critical for precision control.

This critical review systematically examined existing literature on forward and inverse kinematics modeling and IoRT-based robotic control, encompassing analytical, optimization-based, and learning-driven approaches for serial manipulators. Analytical kinematic methods were shown to provide deterministic, computationally efficient solutions but are often treated as isolated mathematical models with limited integration into cloud-enabled or IoRT control architectures. Optimization-based and evolutionary techniques enhance flexibility in constrained and complex environments; however, their iterative nature, parameter sensitivity, and high computational cost restrict their suitability for real-time and deterministic industrial applications. Learning-based inverse kinematics approaches offer fast inference and scalability but suffer from limited interpretability, dataset dependency, and lack of formal feasibility guarantees. Meanwhile, IoRT and cloud robotics studies have primarily focused on connectivity and middleware design, largely neglecting the incorporation of analytical kinematic computation and real-time motion generation, thereby revealing a critical gap in achieving robust, kinematics-aware, and scalable IoRT-based control of industrial robotic manipulators.

Recent developments have extended robotic manipulation beyond classical kinematic modeling through learning-based action selection, cognitive perception, and cyber–physical integration. Phan et al. ^32^ proposed a hierarchical pushing–grasping fusion framework that combines task-specific grasping and pushing masks with reinforcement learning to improve manipulation performance in cluttered environments. The approach was evaluated through simulation and physical experiments, demonstrating the effectiveness of coordinated motion primitives; however, its principal focus is perception-driven task execution rather than analytical inverse kinematics or cloud–edge timing analysis. Nguyen et al. ^33^ subsequently introduced the VLM-RLPGS framework, which integrates vision–language modeling and reinforcement learning to support cognitive push–grasp action selection for industrial manipulators. Numerical simulations and experiments using a UR5 manipulator demonstrated the feasibility of combining visual reasoning with learned manipulation policies, although the method depends on perception, training data, and learning-based decision processes rather than non-iterative closed-form kinematic computation.

From a cyber–physical perspective, Nguyen et al. ^34^ developed a digital-twin-enabled framework for human–robot interaction that integrates real-time data communication, synchronized physical and virtual models, obstacle-aware motion planning, and autonomous manipulation. This architecture provides relevant evidence regarding communication-supported and distributed robotic operation; nevertheless, its primary emphasis is digital-twin-based interaction and motion planning rather than cloud-based multi-branch analytical IK selection and comparative MQTT–REST communication evaluation. Collectively, these studies illustrate the progression toward intelligent and interconnected robotic manipulation while highlighting the complementary role of interpretable analytical kinematics within cloud–edge IoRT architectures.

Table 1 summarizes the reviewed kinematic modeling methods and IoRT-based control approaches.

**Table 1:** Comparative Analysis of Kinematic Modeling Techniques and Their Integration with IoRT.

References	Core idea / method category	Modeling accuracy	Key contributions	Main limitations	Comments
^19^	Analytical IK for redundant S–R–S using virtual arm-angle decoupling	High accuracy analytic IK for 7-DOF redundant manipulator	Closed-form IK (position space), avoids Jacobian/joint-speed; joint-limit strategy	Limited dynamic evaluation; unclear computational comparison/generalizability	Strong analytical formulation but needs clearer real-time + platform validation and broader generalization tests
^20^	Analytical FK via DH + full HTM derivation	Classical DH modeling with precise homogeneous transforms	Full FK derivation + pose extraction + verification plots	Focused on FK only; limited adaptive/networked control discussion	Solid FK reference but does not close the IK + cloud execution gap for industrial control
^21^	Automatic DH extraction from robot geometry	Automated DH parameter extraction with < 4% error	Reduces modeling errors, automates DH parameterization	Still modeling-centric; limited integration with execution/control validation	Useful for reducing setup friction; needs benchmarking against manual DH and downstream FK/IK stability
^22^	CGA-based kinematics representation	CGA-based DH parameter extraction improves modeling elegance	Compact kinematic formulation, alternative to classic matrices	Added math complexity; adoption barrier; limited industrial validation	Promising mathematically but must prove practical benefits vs DH in control pipelines
^23^	GA multi-objective IK/trajectory optimization	DH-based forward kinematics with multi-objective optimization	Balances accuracy/energy/smoothness objectives	Iterative cost + tuning; uncertain real-time suitability	Good constraint handling, but cloud/real-time determinism remains questionable
^24^	Gradient projection + RRT* planning	DH kinematic model with gradient projection and RRT*	Integrates IK feasibility with obstacle-aware planning	Sensitive to initial conditions; computing heavy in cluttered scenes	Strong for obstacle environments; needs timing/latency studies for networked control
^25^	GA + FK/IK + AHP for trajectory decisions	DH-based kinematics with GA for joint path optimization	Multi-criteria decision + trajectory quality improvements	Heuristic dependence; limited deterministic guarantees	Practical planning mix, but hard to guarantee repeatability for industrial execution
^26^	Hybrid metaheuristic IK (IDEA / DE + WOA)	Improved differential evolutionary algorithm for IK	Improved convergence vs single metaheuristics	Still iterative; interpretability + real-time deployment unclear	Good optimizer engineering; still needs deterministic timing and safety constraints for deployment
^27^	PSO / hybrid IK solvers	PSO is the accurate method for kinematic analysis, and RoboAnalyzer the fastest execution time	Robust constraint handling via swarm intelligence	Computation cost; tuning sensitivity; limited determinism	Adds optimization diversity; needs real-world timing and reliability benchmarks
^28^	DNN-based IK (supervised learning)	DH forward kinematics with DNN inverse kinematics	Fast inference after training; smooth outputs	Dataset dependence; unclear generalization/joint-limit guarantees	Good speed, but reliability outside training distribution is the key risk for industry
^29^	Survey/review of deep IK	Review of DNN methods for IK and control	Comprehensive taxonomy of DL IK methods	Highlights issues: interpretability, bias, feasibility guarantees, sim-only validation	Strong review anchor; supports your argument for analytical IK in safety–critical settings
^30^	IoRT middleware for heterogeneous collaboration	Multi-robot / heterogeneous	Improves interoperability and communication architecture	Limited dynamic testing: OS compatibility + latency effects unclear	Strong architecture layer, but kinematics-level integration remains missing
^31^	IoRT robotic arm (cloud/IoT control)	IoRT used 5 DOF Robot Arm controlled by heterogeneous devices via Social IoT and ‘Cloud Robotics’	Early feasibility of cloud/IoT remote control	Limited kinematics integration; weak latency/reliability analysis	Good historical baseline supports your claim that kinematics-aware cloud control is still underdeveloped

## Material and methods

The objective of this study is to investigate cloud-assisted kinematic control of an industrial robotic manipulator by integrating analytical kinematic modeling with an IoRT-enabled layered control framework. As a benchmark platform, the PUMA 560 six-degree-of-freedom (6-DOF) industrial manipulator is selected due to its well-established kinematic structure and suitability for closed-form forward and inverse kinematics analysis. The methodology begins with kinematic derivation using the standard Denavit–Hartenberg (DH) convention, where coordinate frames are assigned to each link and the corresponding DH parameters are used to formulate the homogeneous transformation matrices between successive joints. These transformations are sequentially multiplied to obtain the complete forward kinematics mapping from the base frame to the end-effector frame, yielding the end-effector position and orientation. Based on the derived forward kinematics model and the robot’s spherical wrist structure, a closed-form inverse kinematics formulation is then developed to compute feasible joint configurations for a desired Cartesian pose, accounting for multiple solution branches and joint constraints.

All models are implemented and validated in MATLAB, which is used for symbolic derivation, numerical evaluation, and simulation-based verification. Simulation experiments include pose computation, trajectory execution, and workspace visualization to confirm the correctness and consistency of the analytical kinematic formulations. To evaluate IoRT-enabled operation, a cloud/edge control model is constructed in which the inverse kinematics and solution-selection procedures are executed within a simulated cloud layer, and the resulting joint-space commands are transmitted through a modeled communication interface to an edge/embedded controller responsible for constraint checking and joint-space execution. Feedback of joint states is incorporated to support monitoring and validation of execution behavior under networked conditions.

The proposed framework is assessed using simulation-based evaluation metrics, including (i) kinematic accuracy (pose and trajectory consistency between desired and achieved end-effector motion), (ii) trajectory tracking behavior in joint space and task space, and (iii) timing/response performance of the cloud–edge loop under network-induced delays, reflecting the feasibility of deterministic kinematic computation within IoRT-based robotic control.

### Kinematic analysis using mathematical modeling

Kinematic analysis describes the motion of a robotic manipulator by establishing the mathematical relationship between the joint variables and the resulting position and orientation of the end effector, without considering the forces or torques responsible for generating motion ^35^. This analysis provides the fundamental mapping between joint space and Cartesian space, which is essential for motion planning, trajectory generation, and robotic control. Kinematic analysis is generally divided into forward kinematics, which uniquely determines the end-effector pose from a known set of joint variables, and inverse kinematics, which computes the joint variables required to achieve a desired end-effector pose and is inherently more complex due to nonlinearity, multiple solution branches, and kinematic constraints ^36^.

**Fig. 3:**
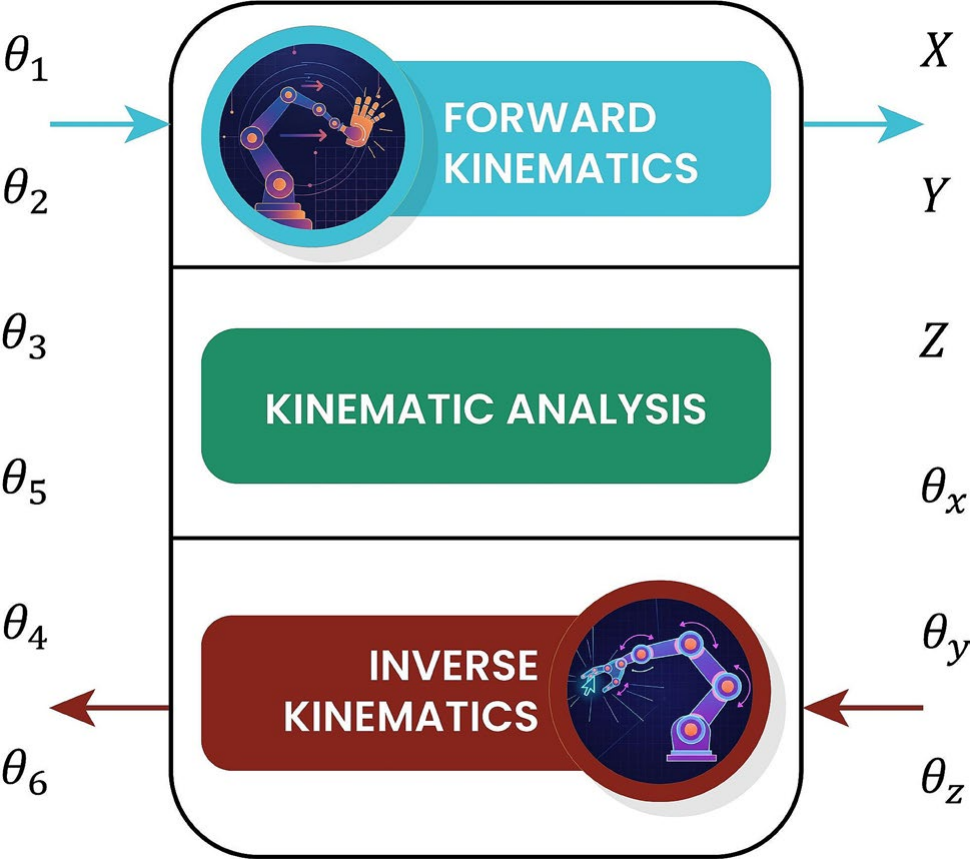
Forward and Inverse kinematics Relationship.

In this study, the six-degree-of-freedom PUMA 560 industrial manipulator is adopted as a case study platform due to its articulated anthropomorphic structure and spherical wrist configuration, which enable the decoupling of position and orientation and facilitate closed-form analytical kinematic solutions. The kinematic model of the manipulator is formulated using the Denavit–Hartenberg (DH) convention, which provides a systematic procedure for assigning coordinate frames to successive links and defining their geometric relationships. Based on this convention, homogeneous transformation matrices are constructed for each joint and subsequently combined to represent the complete kinematic chain from the base frame to the end-effector frame ^37^. These homogeneous transformations offer a unified mathematical representation of rotation and translation, forming the foundation for the analytical derivation of both forward and inverse kinematics within a purely mathematical and simulation-based framework. Such a formulation is particularly suitable for integration into IoRT-enabled cloud-based robotic control architectures, where deterministic and interpretable kinematic models are required to support reliable networked computation and execution ^38^. Figure 3 illustrates the conceptual relationship between forward and inverse kinematics, highlighting the bidirectional mapping between joint space and task space that underpins robotic motion planning and control.

Based on this mathematical kinematic framework, the specific mechanical structure and geometric characteristics of the selected robotic platform must be defined, which are presented in the following section.

#### Robot description and specifications

**Fig. 4:**
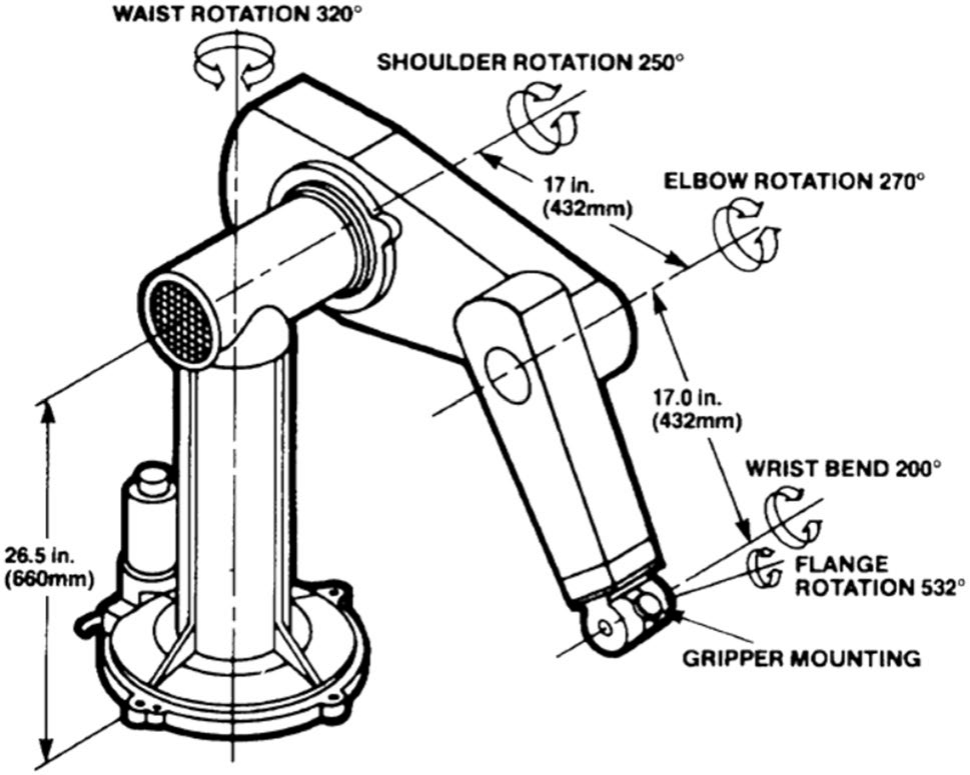
PUMA 560 robotic manipulator.

**Fig. 5:**
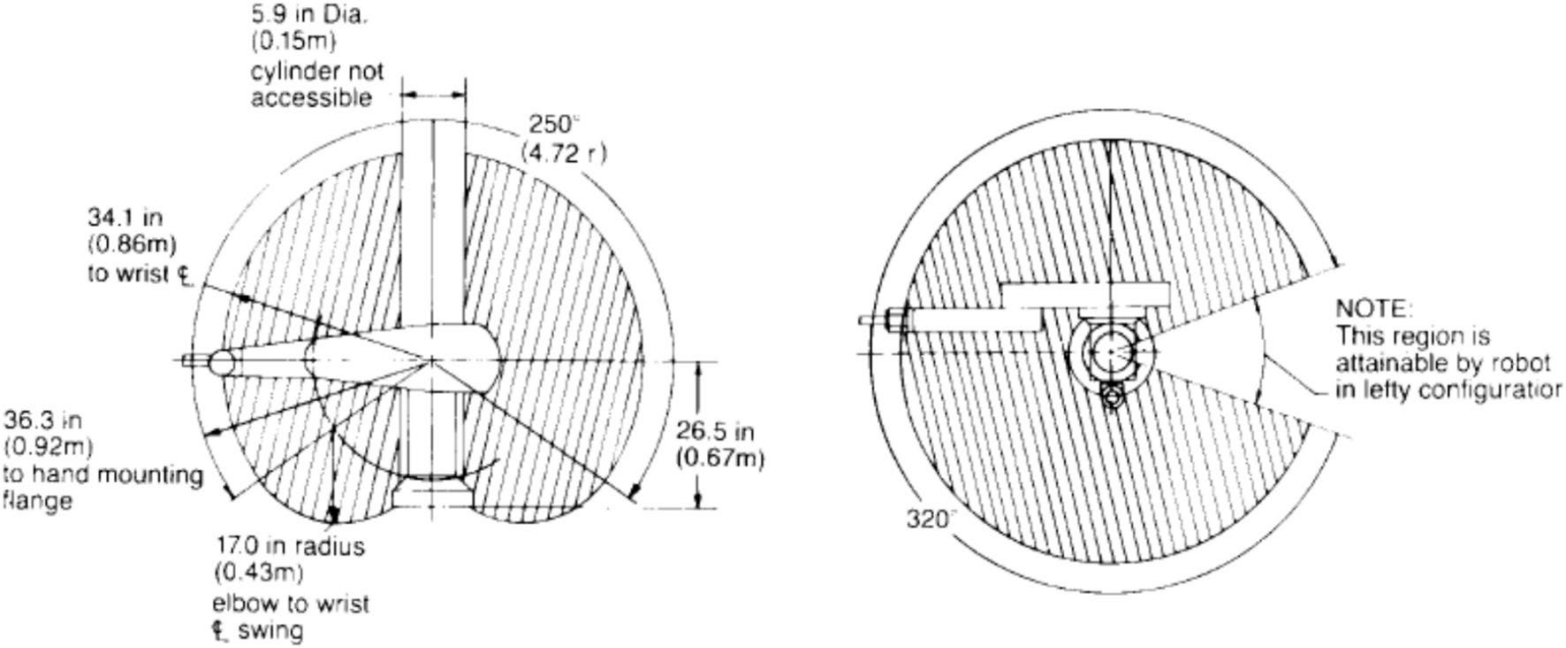
Workspace of PUMA 560 manipulator.

The PUMA 560 is a classical industrial articulated manipulator that has been extensively adopted as a benchmark platform in robotic kinematics and control research. It is a six-degree-of-freedom (6-DOF) serial manipulator composed entirely of revolute joints, enabling full control of the end-effector position and orientation within three-dimensional space ^39^. The mechanical structure consists of an anthropomorphic arm followed by a spherical wrist, in which the rotational axes of the last three joints intersect at a common point. This configuration allows the decoupling of position and orientation in inverse kinematics analysis and makes the PUMA 560 particularly suitable for closed-form analytical solutions. From a kinematic perspective, the first three joints primarily determine the position of the wrist center, while the remaining three joints control the orientation of the end effector ^40^. In this study, the PUMA 560 is modeled using nominal kinematic parameters reported in the literature, and joint variables are constrained within typical industrial angular limits to ensure physically meaningful configurations. Dynamic effects, structural flexibility, and actuator nonlinearities are neglected, as the focus of this work is strictly on kinematic modeling and simulation-based IoRT execution. Figure 4 and Fig. 5 illustrate the PUMA 560 robotic manipulator, highlighting its articulated structure, joint configuration, dimensions, and reachable workspace.

Table 2 summarizes the key kinematic and physical specifications of the PUMA 560 manipulator considered in this study, including DOF, joint limits, workspace characteristics, and payload capacity ^41^.

**Table 2:** Kinematic and Physical Specifications of the PUMA 560 manipulator.

Parameter	Specification
Robot type	Serial articulated industrial manipulator
Degrees of freedom	6 (all revolute joints)
Joint configuration	R–R–R–R–R–R
Wrist structure	Spherical wrist (axes of joints 4–6 intersect)
Typical joint angle limits	J1: ± 160°, J2: − 180° to + 70°, J3: − 45° to + 220°, J4: ± 150°, J5: ± 100°, J6: ± 266°
Maximum horizontal reach	≈ 0.85–1.0 m (depending on configuration)
Workspace shape	Irregular 3D volume defined by serial joint limits
Payload capacity	≈ 2.5 kg
Repeatability (typical)	± 0.1 mm (industrial specification)
Kinematic modeling method	Denavit–Hartenberg (DH) convention
Study scope	Simulation-based Kinematics and IoRT analysis

To formally describe the motion of the PUMA 560 manipulator within this kinematic framework, a consistent assignment of coordinate frames to each link is required, as detailed in the next section.

#### Coordinate frame assignment

Accurate coordinate frame assignment is a fundamental step in the kinematic modeling of serial robotic manipulators, as it establishes the geometric reference needed to describe joint motion and link relationships. In this work, the coordinate frames of the PUMA 560 manipulator are assigned using the standard Denavit–Hartenberg (DH) convention, which provides a systematic and widely accepted methodology for representing articulated kinematic chains. This frame assignment forms the basis for constructing the homogeneous transformation matrices between adjacent links, which are subsequently combined to obtain the overall transformation from the base frame to the end-effector frame, enabling analytical derivation of both forward and inverse kinematics ^42^.

**Fig. 6:**
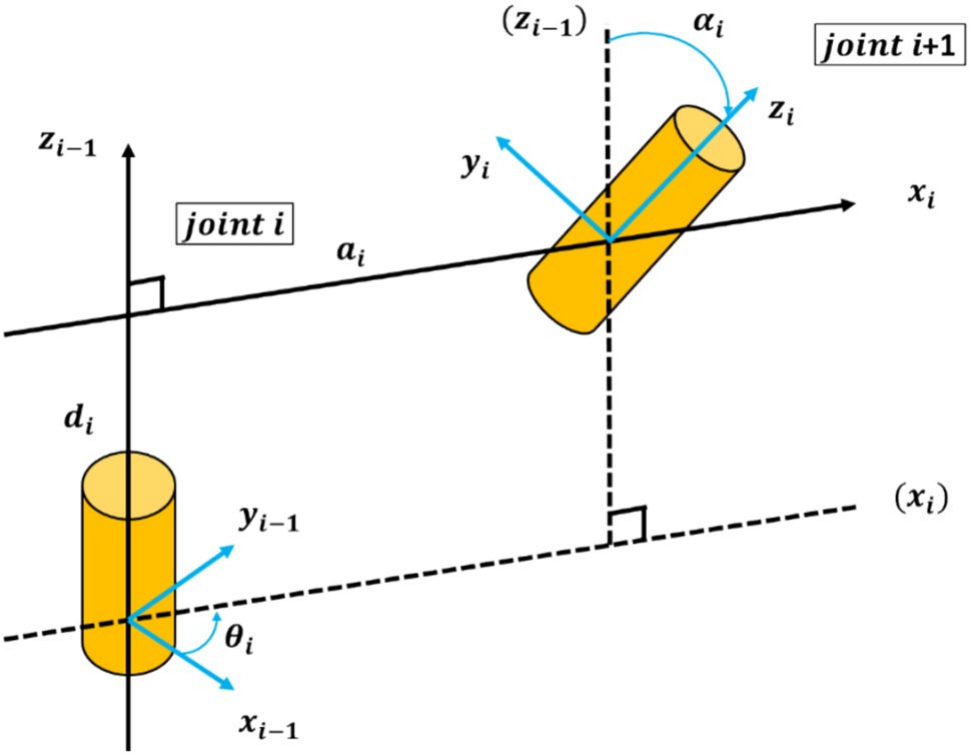
Joint frames assignment.

Following the DH convention, a right-handed coordinate frame $\{i\}$ is attached to each link such that the ${z}_{i}$ axis is aligned with the axis of rotation of joint *i*, while the ${x}_{i}$-axis is oriented along the common normal between consecutive joint axes. The origin of each frame is placed at the intersection of the joint axis and the corresponding common normal, ensuring a consistent geometric interpretation of link parameters. This assignment allows the relative pose between successive frames to be fully described using four DH parameters: the link length ${a}_{i}$, link twist ${\alpha}_{i}$, link offset ${d}_{i}$, and joint angle ${\theta}_{i}$. The frames corresponding to each axis are illustrated in Fig. 6^41^.

**Fig. 7:**
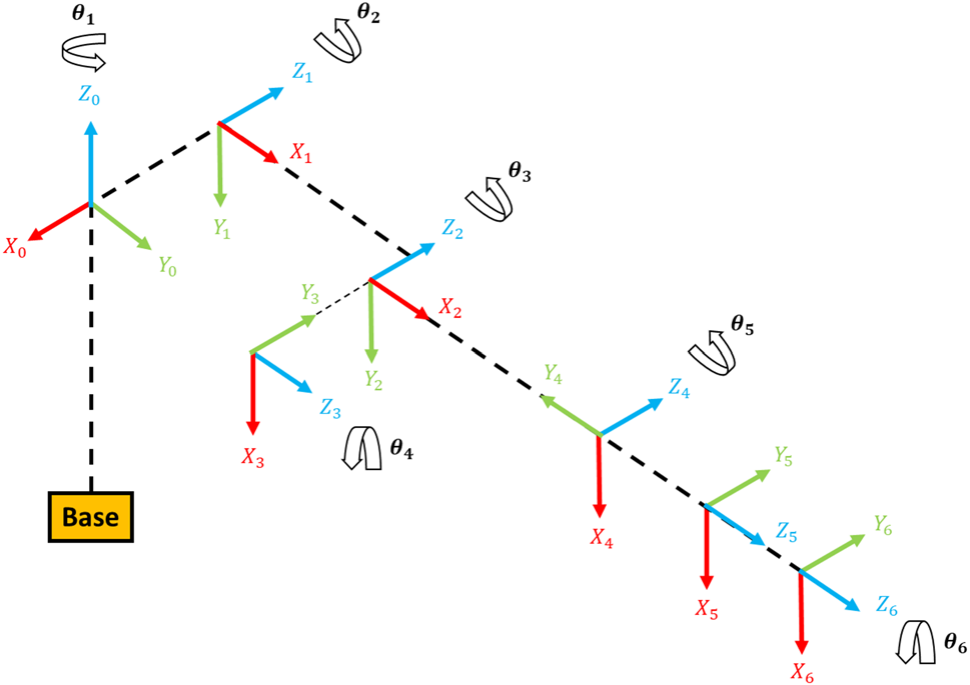
Assignment of joint frames for the PUMA 560 manipulator.

For the PUMA 560, the base coordinate frame $\{0\}$ is fixed at the robot base, and successive frames $\{1\}$ through $\{6\}$ are assigned along the kinematic chain up to the end-effector frame. The first three frames correspond to the anthropomorphic arm responsible for positioning the wrist center, while the last three frames are associated with the spherical wrist that governs end-effector orientation. This arrangement naturally supports the decoupling of position and orientation in inverse kinematics analysis and simplifies the interpretation of both forward and inverse kinematics equations. Figure 7 illustrates the assigned DH coordinate frames for the PUMA 560 robot.

With the coordinate frames systematically assigned according to the DH convention, the corresponding kinematic parameters can now be defined to construct the mathematical model of the manipulator.

#### Kinematic modeling using DH parameters

Following the assignment of coordinate frames, the kinematic model of the PUMA 560 manipulator is formulated using the Denavit–Hartenberg convention, which provides a compact and systematic representation of the geometric relationship between successive links. The DH method describes the spatial transformation between adjacent coordinate frames using four parameters per joint: the link length ${a}_{i}$, link twist ${\alpha}_{i}$, link offset ${d}_{i}$, and joint angle ${\theta}_{i}$. This parameterization enables consistent derivation of homogeneous transformation matrices and facilitates analytical treatment of both forward and inverse kinematics ^43^.

For the PUMA 560 robot, each of the six revolute joints is modeled using a corresponding DH parameter set based on the coordinate frames defined in the previous section. The joint angle ${\theta}_{i}$ represents the only variable parameter for each joint, while the remaining DH parameters are constant and determined by the mechanical design of the manipulator. This formulation captures the anthropomorphic structure of the robot and explicitly reflects the spherical wrist configuration associated with the last three joints. The DH parameters used in this study are listed in Table 3.

**Table 3:** D-H parameters of the PUMA 560 manipulator.

${L}_{i}$	${\theta}_{i}$	${d}_{i}$	${a}_{i}$	${\alpha}_{i}$
1	${\uptheta}_{1}$	*0*	0	90
2	${\uptheta}_{2}$	0	${a}_{2}$	0
3	${\uptheta}_{3}$	${d}_{3}$	${a}_{3}$	-90
4	${\uptheta}_{4}$	${d}_{4}$	0	90
5	${\uptheta}_{5}$	0	0	-90
6	${\uptheta}_{6}$	0	0	0

Using the DH parameters in Table 3, the homogeneous transformation matrix between frame $\{i-1\}$ and frame $\{i\}$ is expressed as a function of the corresponding joint variables and link dimensions. These individual transformations serve as the fundamental building blocks of the complete kinematic chain and are sequentially multiplied to obtain the forward kinematics mapping from the base frame to the end-effector frame. This DH-based kinematic model ensures mathematical consistency, modularity, and direct compatibility with symbolic and numerical computation environments such as MATLAB. By adopting a standardized DH formulation, the kinematic analysis remains transparent, reproducible, and well suited for integration within the proposed IoRT-enabled simulation framework.

To formally describe the kinematic relationship between two consecutive links, the homogeneous transformation from frame $\{i-1\}$ to frame $\{i\}$ is defined as the ordered sequence of elementary rotations and translations prescribed by the Denavit–Hartenberg convention:

1 $${A}_{i}={Rot }_{z,{\theta}_{i}}{Trans}_{z,{d}_{i} }{Trans}_{x,{a}_{i} }{Rot }_{x,{\alpha}_{i}}$$

For a given link *i*, the corresponding homogeneous transformation matrix can be written explicitly as the product of four elementary transformation matrices:

2 $${A}_{i}=\left[\begin{array}{cccc}\mathit{cos}{\theta}_{i}& -\mathit{sin}{\theta}_{i}\mathit{cos}{\alpha}_{i}& \mathit{sin}{\theta}_{i}\mathit{sin}{\alpha}_{i}& {a}_{i}\mathit{cos}{\theta}_{i}\\ \mathit{sin}{\theta}_{i}& \mathit{cos}{\theta}_{i}\mathit{cos}{\alpha}_{i}& -\mathit{cos}{\theta}_{i}\mathit{sin}{\alpha}_{i}& {a}_{i}\mathit{sin}{\theta}_{i}\\ 0& \mathit{sin}{\alpha}_{i}& \mathit{cos}{\alpha}_{i}& {d}_{i}\\ 0& 0& 0& 1\end{array}\right]$$

The homogeneous transformation matrix in Eq. (2) represents the fundamental building block of the robot’s kinematic chain. By sequentially multiplying the individual transformation matrices ${A}_{i}$ for *i=1,… ,6*, the complete forward kinematics mapping from the base frame to the end-effector frame of the PUMA 560 manipulator is obtained, as detailed in the following section.

### Forward kinematics

Forward kinematics describes the mathematical process of determining the position and orientation of a robot’s end effector in the task space given a known set of joint variables. For serial robotic manipulators, forward kinematics establishes a deterministic mapping from joint space to Cartesian space and provides a unique solution for each joint configuration. This makes forward kinematics a fundamental tool for robot simulation, motion visualization, workspace analysis, and verification of inverse kinematics solutions ^44^.

In the case of the PUMA 560 six-degree-of-freedom manipulator, the forward kinematics problem is formulated to compute the complete pose of the end effector, including both translational position and rotational orientation with respect to the base coordinate frame. Accurate forward kinematics is essential for validating the correctness of the inverse kinematics solutions and for evaluating the feasibility of desired end-effector trajectories within the robot’s reachable workspace ^45^.

To determine the pose of the end effector, transformation matrices for all six joints must be calculated. This process begins by independently computing each coordinate transformation using Eq. (2), with the values from Table 3 substituted into the equation. The kinematic analysis starts with the first joint, located at the base of the robot, and proceeds sequentially through the second joint, third joint, and so forth until the final analysis of the end effector is completed ^46^. The transformation matrices for the six joints are computed as indicated from Eqs. (3) to (8). The analytical formulation of forward kinematics is implemented and evaluated in MATLAB.

3 $${A}_{1}^{0}=\left[\begin{array}{cccc}\mathit{cos}{\theta}_{1}& 0& \mathit{sin}{\theta}_{1}& 0\\ \mathit{sin}{\theta}_{1}& 0& -\mathit{cos}{\theta}_{1}& 0\\ 0& 1& 0& 0\\ 0& 0& 0& 1\end{array}\right]$$

4 $${A}_{2}^{1}=\left[\begin{array}{cccc}\mathit{cos}{\theta}_{2}& -\mathit{sin}{\theta}_{2}^{ }& 0& { a}_{2}\mathit{cos}{\theta}_{2}\\ \mathit{sin}{\theta}_{2}^{ }& \mathit{cos}{\theta}_{2}& 0& { a}_{2}\mathit{sin}{\theta}_{2}^{ }\\ 0& 0& 1& 0\\ 0& 0& 0& 1\end{array}\right]$$

5 $${A}_{3}^{2}=\left[\begin{array}{cccc}\mathit{cos}{\theta}_{3}& 0& -\mathit{sin}{\theta}_{3}^{ }& { a}_{3}\mathit{cos}{\theta}_{3}\\ \mathit{sin}{\theta}_{3}^{ }& 0& \mathit{cos}{\theta}_{3}& { a}_{3}\mathit{sin}{\theta}_{3}^{ }\\ 0& -1& 0& {d}_{3}\\ 0& 0& 0& 1\end{array}\right]$$

6 $${A}_{4}^{3}=\left[\begin{array}{cccc}\mathit{cos}{\theta}_{4}& 0& \mathit{sin}{\theta}_{4}^{ }& 0\\ \mathit{sin}{\theta}_{4}^{ }& 0& -\mathit{cos}{\theta}_{4}& 0\\ 0& 1& 0& {d}_{4}\\ 0& 0& 0& 1\end{array}\right]$$

7 $${A}_{5}^{4}=\left[\begin{array}{cccc}\mathit{cos}{\theta}_{5}& 0& -\mathit{sin}{\theta}_{5}^{ }& 0\\ \mathit{sin}{\theta}_{5}^{ }& 0& \mathit{cos}{\theta}_{5}& 0\\ 0& -1& 0& 0\\ 0& 0& 0& 1\end{array}\right]$$

8 $${A}_{6}^{5}=\left[\begin{array}{cccc}\mathit{cos}{\theta}_{6}^{ }& -\mathit{sin}{\theta}_{6}^{ }& 0& 0\\ \mathit{sin}{\theta}_{6}^{ }& \mathit{cos}{\theta}_{6}^{ }& 0& 0\\ 0& 0& 1& 0\\ 0& 0& 0& 1\end{array}\right]$$

9 $${T}_{6}^{0}=\left[\begin{array}{cccc}{n}_{x}& {o}_{x}& {a}_{x}& {p}_{x}\\ {n}_{y}& {o}_{y}& {a}_{y}& {p}_{y}\\ {n}_{z}& {o}_{z}& {a}_{z}& {p}_{z}\\ 0& 0& 0& 1\end{array}\right]= {A}_{1}^{0} \times {A}_{2}^{1} \times {A}_{3}^{2} \times {A}_{4}^{3} \times {A}_{5}^{4} \times {A}_{6}^{5}$$

Equation (9) present the total transformation matrix of the robot ${T}_{6}^{0}$. The Puma 560 robot’s forward kinematics equations are determined as indicated from Eq. (9) to Eq. (21). The orientation of the manipulator end effector is represented by ${n}_{x}$,${n}_{y}$,${n}_{z}$,${o}_{x}$,${o}_{y}$, ${o}_{z}$,${a}_{x}$,${a}_{y}$, and ${a}_{z}$ while the position of the end effector is represented by ${p}_{x}$,${p}_{y}$, and ${p}_{z}$. The position and orientation of the end effector are function of ${\theta}_{1}$, ${\theta}_{2}$, ${\theta}_{3}$, ${\theta}_{4}$, ${\theta}_{5}$ and ${\theta}_{6}$.

10 $$\begin{gathered} \boldsymbol{n}_{\boldsymbol{x}} = - ~cos\theta _{6} \left( {\left( {sin\theta _{1} sin\theta _{4} - cos\theta _{1} cos\theta _{4} \left( { - sin\theta _{2} sin\theta _{3} + cos\theta _{2} cos\theta _{3} } \right)} \right)cos\theta _{5} + \left( {sin\theta _{2} cos\theta _{3} + sin\theta _{3} cos\theta _{2} } \right)cos\theta _{1} sin\theta _{5} } \right) \hfill \\ \quad - sin\theta _{6} \left( {sin\theta _{1} cos\theta _{4} + sin\theta _{4} cos\theta _{1} \left( { - sin\theta _{2} sin\theta _{3} + cos\theta _{2} cos\theta _{3} } \right)} \right) \hfill \\ \end{gathered}$$

11 $$\begin{gathered} \boldsymbol{n}_{\boldsymbol{y}} = cos\theta _{6} \left( {\left( {sin\theta _{1} cos\theta _{4} \left( { - sin\theta _{2} sin\theta _{3} + cos\theta _{2} cos\theta _{3} } \right) + sin\theta _{4} cos\theta _{1} } \right)cos\theta _{5} - \left( {sin\theta _{2} cos\theta _{3} + sin\theta _{3} cos\theta _{2} } \right)sin\theta _{1} sin\theta _{5} } \right) \hfill \\ \quad - sin\theta _{6} \left( {sin\theta _{1} sin\theta _{4} \left( { - sin\theta _{2} sin\theta _{3} + cos\theta _{2} cos\theta _{3} } \right) - cos\theta _{1} cos\theta _{4} } \right) \hfill \\ \end{gathered}$$

12 $$\begin{gathered} \boldsymbol{n}_{\boldsymbol{z}} = - cos\theta _{6} \left( {\left( { - sin\theta _{2} sin\theta _{3} + cos\theta _{2} cos\theta _{3} } \right)sin\theta _{5} + \left( {sin\theta _{2} cos\theta _{3} + sin\theta _{3} cos\theta _{2} } \right)cos\theta _{4} cos\theta _{5} } \right) \hfill \\ \quad - sin\theta _{4} sin\theta _{6} \left( {sin\theta _{2} cos\theta _{3} + sin\theta _{3} cos\theta _{2} } \right) \hfill \\ \end{gathered}$$

13 $$\begin{gathered} \boldsymbol{o}_{\boldsymbol{x}} = - sin\theta _{6} \left( {\left( {sin\theta _{1} sin\theta _{4} - cos\theta _{1} cos\theta _{4} \left( { - sin\theta _{2} sin\theta _{3} + cos\theta _{2} cos\theta _{3} } \right)} \right)cos\theta _{5} + \left( {sin\theta _{2} cos\theta _{3} + sin\theta _{3} cos\theta _{2} } \right)cos\theta _{1} sin\theta _{5} } \right) \hfill \\ \quad - cos\theta _{6} \left( {sin\theta _{1} cos\theta _{4} + sin\theta _{4} cos\theta _{1} \left( { - sin\theta _{2} sin\theta _{3} + cos\theta _{2} cos\theta _{3} } \right)} \right) \hfill \\ \end{gathered}$$

14 $$\begin{gathered} \boldsymbol{o}_{\boldsymbol{y}} = - sin\theta _{6} \left( {\left( {sin\theta _{1} cos\theta _{4} \left( { - sin\theta _{2} sin\theta _{3} + cos\theta _{2} cos\theta _{3} } \right) + sin\theta _{4} cos\theta _{1} } \right)cos\theta _{5} - \left( {sin\theta _{2} cos\theta _{3} + sin\theta _{3} cos\theta _{2} } \right)sin\theta _{1} sin\theta _{5} } \right) \hfill \\ \quad - cos\theta _{6} \left( {sin\theta _{1} sin\theta _{4} \left( { - sin\theta _{2} sin\theta _{3} + cos\theta _{2} cos\theta _{3} } \right) - cos\theta _{1} cos\theta _{4} } \right) \hfill \\ \end{gathered}$$

15 $${{\boldsymbol{o}}}_{{\boldsymbol{z}}}=-sin{\theta}_{6}\left(\left(-sin{\theta}_{2}sin{\theta}_{3}+cos{\theta}_{2}cos{\theta}_{3}\right)sin{\theta}_{5}+\left(sin{\theta}_{2}cos{\theta}_{3}+sin{\theta}_{3}cos{\theta}_{2}\right)cos{\theta}_{4}cos{\theta}_{5}\right)-sin{\theta}_{4}cos{\theta}_{6}\left(sin{\theta}_{2}cos{\theta}_{3}+sin{\theta}_{3}cos{\theta}_{2}\right)$$

16 $${{\boldsymbol{a}}}_{{\boldsymbol{x}}}=-sin{\theta}_{5}\left(sin{\theta}_{1}sin{\theta}_{4}-cos{\theta}_{1}cos{\theta}_{4}\left(-sin{\theta}_{2}sin{\theta}_{3}+cos{\theta}_{2}cos{\theta}_{3}\right)\right)+cos{\theta}_{1}cos{\theta}_{5}\left(sin{\theta}_{2}cos{\theta}_{3}+sin{\theta}_{3}cos{\theta}_{2}\right)$$

17 $${{\boldsymbol{a}}}_{{\boldsymbol{y}}}=-sin{\theta}_{5}\left(sin{\theta}_{1}cos{\theta}_{4}\left(-sin{\theta}_{2}sin{\theta}_{3}+cos{\theta}_{2}cos{\theta}_{3}\right)+sin{\theta}_{4}cos{\theta}_{1}\right)+sin{\theta}_{1}cos{\theta}_{5}\left(sin{\theta}_{2}cos{\theta}_{3}+sin{\theta}_{3}cos{\theta}_{2}\right)$$

18 $${{\boldsymbol{a}}}_{{\boldsymbol{z}}}=cos{\theta}_{5}\left(-sin{\theta}_{2}sin{\theta}_{3}+cos{\theta}_{2}cos{\theta}_{3}\right)-cos{\theta}_{4}sin{\theta}_{5}\left(sin{\theta}_{2}cos{\theta}_{3}+sin{\theta}_{3}cos{\theta}_{2}\right)$$

19 $${{\boldsymbol{p}}}_{{\boldsymbol{x}}}={a}_{2}cos{\theta}_{1}cos{\theta}_{2}+{a}_{3}cos{\theta}_{1}\left(-sin{\theta}_{2}sin{\theta}_{3}+cos{\theta}_{2}cos{\theta}_{3}\right)-{d}_{4}\left(sin{\theta}_{2}cos{\theta}_{3}+sin{\theta}_{3}cos{\theta}_{2}\right)cos{\theta}_{1}+{d}_{3}sin{\theta}_{1}$$

20 $${{\boldsymbol{p}}}_{{\boldsymbol{y}}}={a}_{2}sin{\theta}_{1}cos{\theta}_{2}+{a}_{3}sin{\theta}_{1}\left(-sin{\theta}_{2}sin{\theta}_{3}+cos{\theta}_{2}cos{\theta}_{3}\right)-{d}_{3}cos{\theta}_{1}-{d}_{4}\left(sin{\theta}_{2}cos{\theta}_{3}+sin{\theta}_{3}cos{\theta}_{2}\right)sin{\theta}_{1}$$

21 $${{\boldsymbol{p}}}_{{\boldsymbol{z}}}={a}_{2}sin{\theta}_{2}+{a}_{3}\left(sin{\theta}_{2}cos{\theta}_{3}+sin{\theta}_{3}cos{\theta}_{2}\right)+{d}_{4}\left(-sin{\theta}_{2}sin{\theta}_{3}+cos{\theta}_{2}cos{\theta}_{3}\right)$$

**Fig. 8:**
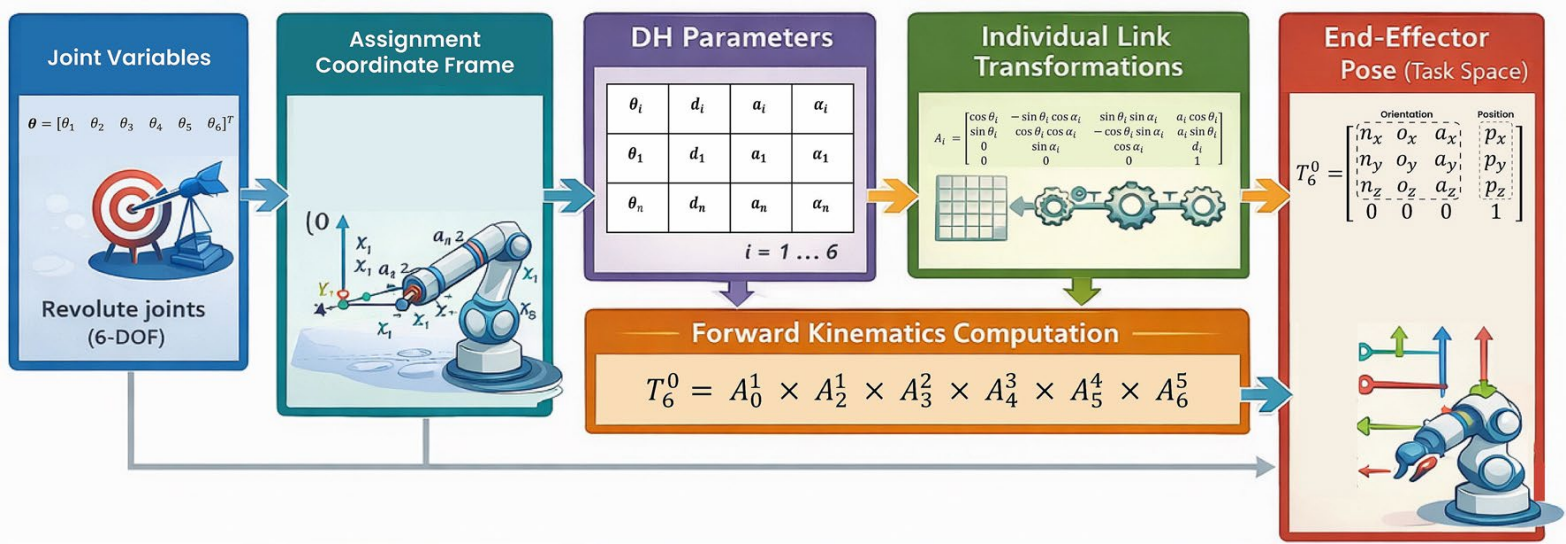
Forward Kinematics Computation Flow.

Figure 8 illustrates the conceptual forward kinematics computational flow of the 6-DOF robot arm.

Using the established DH parameters and homogeneous transformation matrices, the forward kinematics of the PUMA 560 manipulator can now be derived to compute the complete end-effector pose.

### Inverse kinematics

Inverse kinematics is a fundamental problem in robotic manipulation that involves determining the joint variables required to achieve a specified end-effector pose in Cartesian space. Unlike forward kinematics, which provides a unique mapping from joint space to task space, inverse kinematics is generally nonlinear and may admit multiple solutions, a single solution, or no solution depending on the manipulator geometry and the desired pose ^27^. A rigorous inverse kinematics formulation is essential for motion planning, trajectory generation, and real-time control, particularly for redundant or multi-degree-of-freedom robotic systems. In this work, an analytical inverse kinematics solution is derived for the considered 6-DOF manipulator using a geometric decoupling approach enabled by its kinematic structure ^37^. The desired end-effector pose with respect to the base frame is defined as

22 $${T}_{6}^{0}=\left[\begin{array}{cc}{R}_{6}^{0}& p\\ 0& 1\end{array}\right]$$

where ${R}_{6}^{0}$ is the desired orientation matrix and *p* is the desired position vector. The rotation matrix and position vector are given by

23 $${R}_{6}^{0}=\left[\begin{array}{lll}{n}_{x}& {o}_{x}& {a}_{x}\\ {n}_{y}& {o}_{y}& {a}_{y}\\ {n}_{z}& {o}_{z}& {a}_{z}\end{array}\right], P=\left[\begin{array}{c}{p}_{x}\\ {p}_{y}\\ {p}_{z}\end{array}\right]$$

The manipulator is described using the updated standard Denavit–Hartenberg parameters listed in Table 3. Because the last three joints satisfy ${a}_{5}={a}_{6}=0$ and ${d}_{5}={d}_{6}=0$, the wrist axes intersect at a single point, forming a spherical wrist. This property allows the inverse kinematics problem to be decoupled into a position subproblem, which determines ${\theta}_{1},{\theta}_{2},{\theta}_{3}$ from the position vector ${p}$ *and an orientation* subproblem, which determines ${\theta}_{4},{\theta}_{5},{\theta}_{6}$ from the rotation matrix ${R}_{6}^{0}$. Since ${d}_{6}=0$, the wrist center coincides with the end-effector position, yielding

24 $${p}_{c}=p$$

The projection of the end-effector position onto the base plane is defined as

25 $$r=\sqrt{{p}_{x}^{2}+{p}_{y}^{2}}$$

The Cartesian coordinates ${p}_{x}$ and ${p}_{y}$ can be expressed as

26 $${p}_{x}=cos{\theta}_{1}X+{d}_{3}sin{\theta}_{1}$$

27 $${p}_{y}=sin{\theta}_{1}X-{d}_{3}cos{\theta}_{1}$$

where *X* depends only on ${\theta}_{2},{\theta}_{3}$, and the manipulator parameters. Squaring and adding (6) and (7) yields

28 $${p}_{x}^{2}+{p}_{y}^{2}={X}^{2}+{d}_{3}^{2}$$

29 $$X=\pm \sqrt{{r}^{2}-{d}_{3}^{2}}$$

Equations (26) and (27) represent a planar rotation, which can be written in matrix form as

30 $$\left[\begin{array}{l}{p}_{x}\\ {p}_{y}\end{array}\right]=\left[\begin{array}{cc}cos{\theta}_{1}& -sin{\theta}_{1}\\ sin{\theta}_{1}& cos{\theta}_{1}\end{array}\right]\left[\begin{array}{c}X\\ -{d}_{3}\end{array}\right]$$

Therefore, the base joint angle is obtained as

31 $${\theta}_{1}=\mathrm{a}\mathrm{t}\mathrm{a}\mathrm{n}2\left({p}_{y},{p}_{x}\right)+\mathrm{a}\mathrm{t}\mathrm{a}\mathrm{n}2\left({d}_{3},X\right)$$

The two possible values of *X* correspond to the left-arm and right-arm configurations. After computing ${\theta}_{1}$, the end-effector position is rotated into the plane of joints two and three as

32 $$X=cos{\theta}_{1}{p}_{x}+sin{\theta}_{1}{p}_{y}$$

33 $$Z={p}_{z}$$

The equivalent link length and offset angle are defined as

34 $$L=\sqrt{{a}_{3}^{2}+{d}_{4}^{2}}$$

35 $$\gamma =\mathrm{a}\mathrm{t}\mathrm{a}\mathrm{n}2\left({d}_{4},{a}_{3}\right)$$

An auxiliary elbow angle is introduced as

36 $${\theta}_{3}{\prime}={\theta}_{3}+\gamma$$

The distance from joint two to the wrist center is given by

37 $$\rho =\sqrt{{X}^{2}+{Z}^{2}}$$

Applying the law of cosines yields

38 $$\mathrm{c}\mathrm{o}\mathrm{s}{\theta}_{3}{\prime}=\frac{{\rho }^{2}-{a}_{2}^{2}-{L}^{2}}{2{a}_{2}L}$$

This equation admits two solutions corresponding to elbow-up and elbow-down configurations, with

39 $$sin{\theta}_{3}{\prime}=\pm \sqrt{1-{\mathrm{cos}}^{2}{\theta}_{3}{\prime}}$$

40 $${\theta}_{3}{\prime}=atan2\left(sin{\theta}_{3}{\prime},\mathrm{c}\mathrm{o}\mathrm{s}{\theta}_{3}{\prime}\right)$$

and the actual elbow joint angle is recovered as

41 $${\theta}_{3}={\theta}_{3}{\prime}-\gamma$$

The shoulder joint angle is computed by defining

42 $$\phi =\mathrm{a}\mathrm{t}\mathrm{a}\mathrm{n}2\left(Z,X\right)$$

43 $$\psi =\mathrm{a}\mathrm{t}\mathrm{a}\mathrm{n}2\left(L\mathrm{sin}{\theta}_{3}{\prime},{a}_{2}+L\mathrm{cos}{\theta}_{3}{\prime}\right)$$

44 $${\theta}_{2}=\phi -\psi$$

Equations (31), (41), and (44) provide the complete analytical solution for the position subproblem, resulting in up to four configurations. The orientation subproblem is solved using the rotational relationship

45 $${R}_{6}^{0}={R}_{3}^{0}{R}_{6}^{3} \mathrm{w}\mathrm{h}\mathrm{i}\mathrm{c}\mathrm{h} \mathrm{g}\mathrm{i}\mathrm{v}\mathrm{e}\mathrm{s} {R}_{6}^{3}={\left({R}_{3}^{0}\right)}^{T}{R}_{6}^{0}$$

The matrix ${R}_{3}^{0}$ is computed directly from the forward kinematics of the first three joints. The resulting matrix ${R}_{6}^{3}$ contains only the wrist joint variables and is written as

46 $${R}_{6}^{3}=\left[\begin{array}{lll}{r}_{11}& {r}_{12}& {r}_{13}\\ {r}_{21}& {r}_{22}& {r}_{23}\\ {r}_{31}& {r}_{32}& {r}_{33}\end{array}\right]$$

From the wrist kinematic structure, the following relations hold

47 $${r}_{13}=-sin{\theta}_{5}cos{\theta}_{4}$$

48 $${r}_{23}=-sin{\theta}_{5}sin{\theta}_{4}$$

49 $${r}_{33}=cos{\theta}_{5}$$

50 $${r}_{31}=sin{\theta}_{5}cos{\theta}_{6}$$

51 $${r}_{32}=-sin{\theta}_{5}sin{\theta}_{6}$$

Accordingly, the wrist joint angles are obtained as

52 $${\theta}_{5}=atan2\left(\sqrt{{r}_{13}^{2}+{r}_{23}^{2}},{r}_{33}\right)$$

53 $${\theta}_{4}=\mathrm{a}\mathrm{t}\mathrm{a}\mathrm{n}2\left({r}_{23},{r}_{13}\right)$$

54 $${\theta}_{6}=\mathrm{a}\mathrm{t}\mathrm{a}\mathrm{n}2\left(-{r}_{32},{r}_{31}\right)$$

A wrist-flip solution is obtained by

55 $${\theta}_{5}^{\star }=-{\theta}_{5}$$

56 $${\theta}_{4}^{\star }={\theta}_{4}+\pi$$

57 $${\theta}_{6}^{\star }={\theta}_{6}+\pi$$

Combining the arm, elbow, and wrist solutions results in up to eight inverse kinematics solutions for a single end-effector pose. Despite the existence of a closed-form inverse kinematics solution, certain configurations lead to kinematic singularities, where one or more joint variables become indeterminate or the manipulator loses a degree of freedom. In the considered PUMA-type manipulator, singularities primarily arise from the spherical wrist structure. The most critical case occurs when the wrist pitch angle satisfies

58 $$sin{\theta}_{5}=0 , {\theta}_{5}=0 or \pi$$

Under this condition, the rotational axes of joints 4 and 6 become collinear, resulting in a wrist singularity. Consequently, the individual values of ${\theta}_{4}$ and ${\theta}_{6}$ cannot be uniquely determined, and only their sum remains observable from the orientation matrix ${R}_{6}^{3}$. To handle this situation, the following strategy is adopted:

- ${\theta}_{5}$ is fixed to zero (or *π* ) according to the sign of ${r}_{6}^{3}$.
- The combined angle ${\theta}_{4}+{\theta}_{6}$ is extracted from the remaining non-zero elements of ${R}_{6}^{3}$.
- One joint variable (typically ${\theta}_{4}$ ) is assigned a nominal or previously valid value, while the other ( ${\theta}_{6}$ ) is computed accordingly.

This approach preserves orientation continuity and prevents numerical instability in real-time control. Additionally, configurations in which $\left|\mathrm{s}\mathrm{i}\mathrm{n}\left({\theta}_{5}\right)\right|$ falls below a predefined threshold are avoided during trajectory planning to reduce sensitivity near singular postures. The inverse kinematics formulation yields up to eight feasible solutions, corresponding to combinations of left/right arm configurations, elbow up/down postures, and wrist flip/non-flip orientations. However, not all solutions are suitable for execution in practical robotic systems. Therefore, a systematic solution-selection policy is employed to identify the most appropriate configuration. The selection process follows these criteria:

- Joint-Limit Feasibility: All candidate solutions are first checked against the mechanical joint limits of the manipulator. Any solution violating joint constraints is discarded.
- Singularity Avoidance: Solutions that place the manipulator close to wrist or arm singularities ($\left|\mathrm{s}\mathrm{i}\mathrm{n}\left({\theta}_{5}\right)\right|\approx 0$ ) are penalized or rejected to ensure numerical robustness.
- Continuity and Smoothness: Among the remaining solutions, the configuration that minimizes the joint displacement with respect to the previous robot state is selected ^47^:
  59 $${q}^{*}=arg\underset{q}{min} \Vert q-{q}_{prev}\Vert$$
- Task-Specific Optimization: Additional criteria such as obstacle avoidance, or manipulability maximization can be incorporated depending on task requirements.

**Fig. 9:**
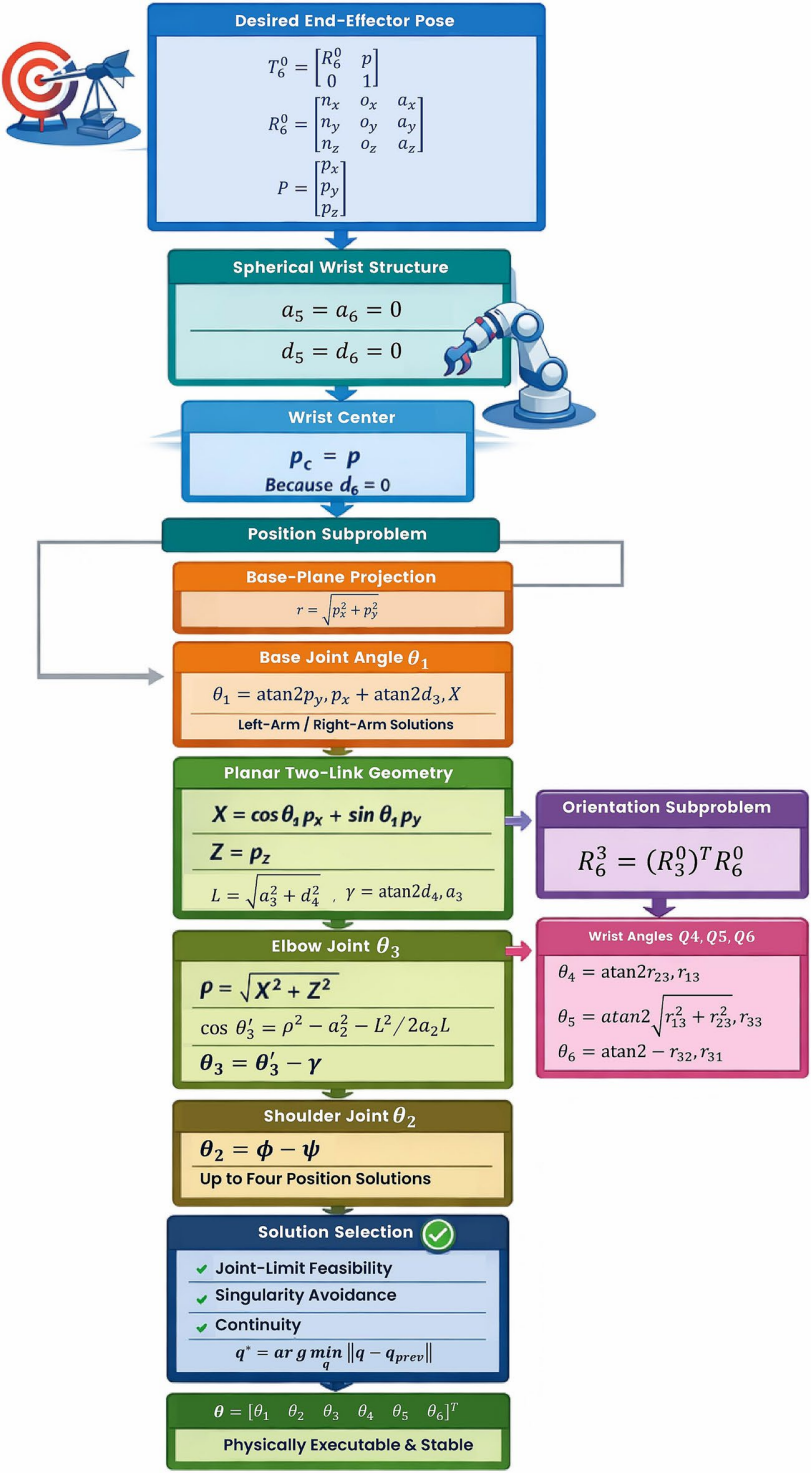
Inverse kinematics solution-selection flow of the 6-DOF manipulator.

This selection policy ensures that the inverse kinematics solution is not only mathematically valid but also physically executable, stable, and suitable for real-time deployment, particularly in networked and IoRT-based robotic control frameworks. Figure 9 summarizes the cloud-based workflow used to generate, filter, evaluate, and select among the discrete analytical inverse-kinematics branches of the 6-DOF manipulator.

Having established an analytical solution for both forward and inverse kinematics, the following section presents the proposed IoRT-based robotic control framework that integrates these kinematic computations within a cloud–edge architecture.

### IoRT-based robotic control framework

The Internet of Robotic Things (IoRT) represents a paradigm shift in robotic system design by enabling robots to operate as intelligent, networked entities that integrate sensing, computation, communication, and control across distributed environments. By leveraging cloud and edge computing infrastructures, IoRT-enabled robotic systems extend computational capabilities, support remote supervision, and enable scalable collaborative operation. This paradigm is particularly relevant for industrial manipulators, where increasing task complexity, real-time monitoring demands, and intelligent decision-making requirements exceed the capabilities of traditional standalone embedded controllers. Despite these advantages, most existing IoRT and cloud robotics frameworks primarily emphasize communication middleware, data exchange, or high-level planning, while fundamental kinematic modeling and inverse kinematics computation remain largely confined to local hardware, limiting scalability and flexibility in distributed robotic control ^30^.

To overcome these limitations, this study proposes an IoRT-based robotic control framework that explicitly integrates analytical forward and inverse kinematics within a cloud-assisted control loop, while preserving local execution safety and real-time responsiveness. Deterministic kinematic computations are offloaded to the cloud to enable centralized motion generation, remote supervision, and scalable control without compromising reliability in industrial environments. The proposed framework adopts a layered IoRT architecture that clearly separates perception, computation, communication, and execution responsibilities between cloud and edge components, ensuring bounded computational latency, full interpretability, and predictable system behavior. In the following sections, the proposed IoRT-based control framework is evaluated through analytical validation and experimental performance assessment to demonstrate its correctness, timing feasibility, and effectiveness in cloud-assisted robotic manipulation ^18^.

#### IoRT-based layered control architecture

**Fig. 10:**
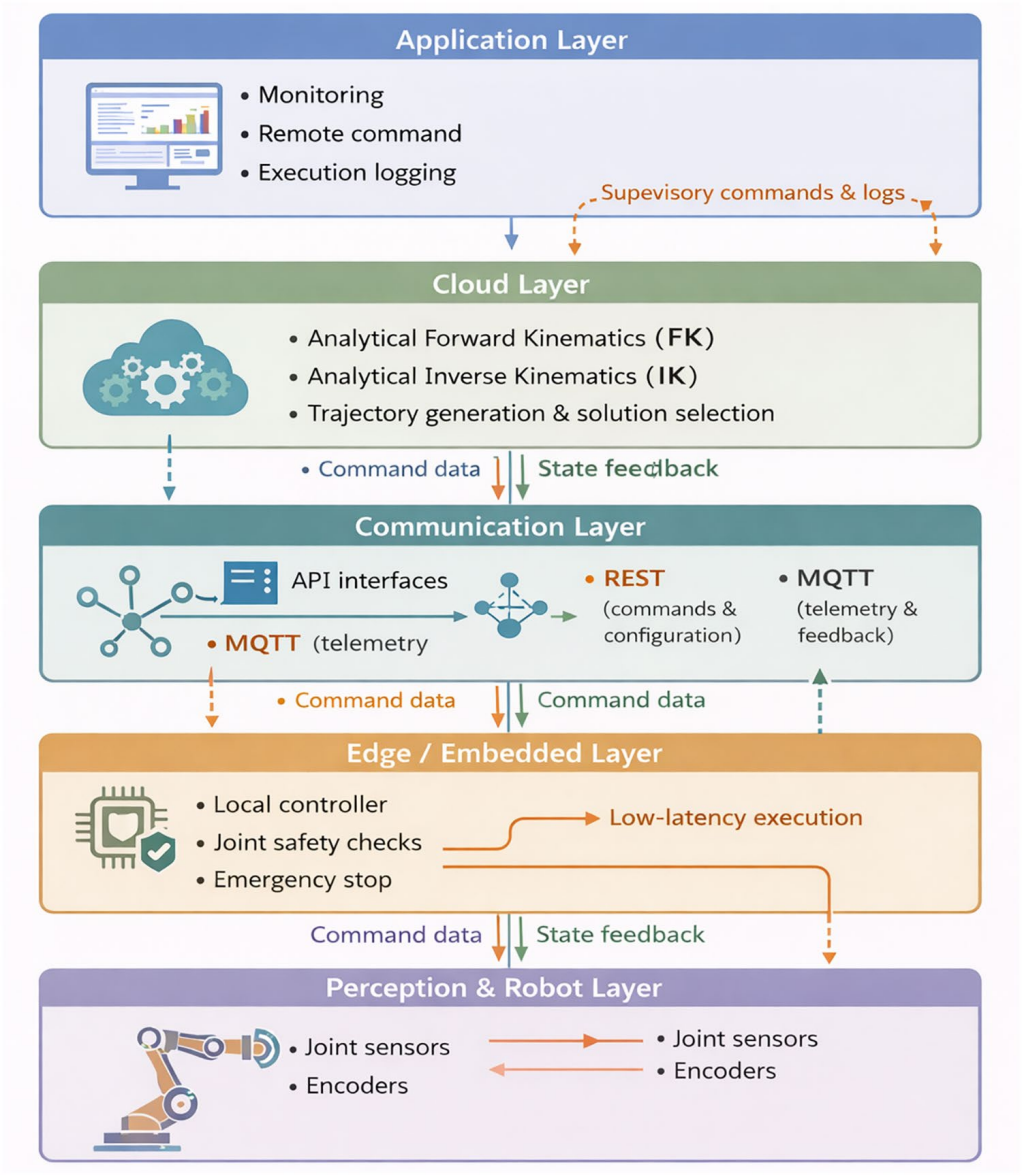
Layered IoRT-based robotic control architecture.

**Fig. 11:**
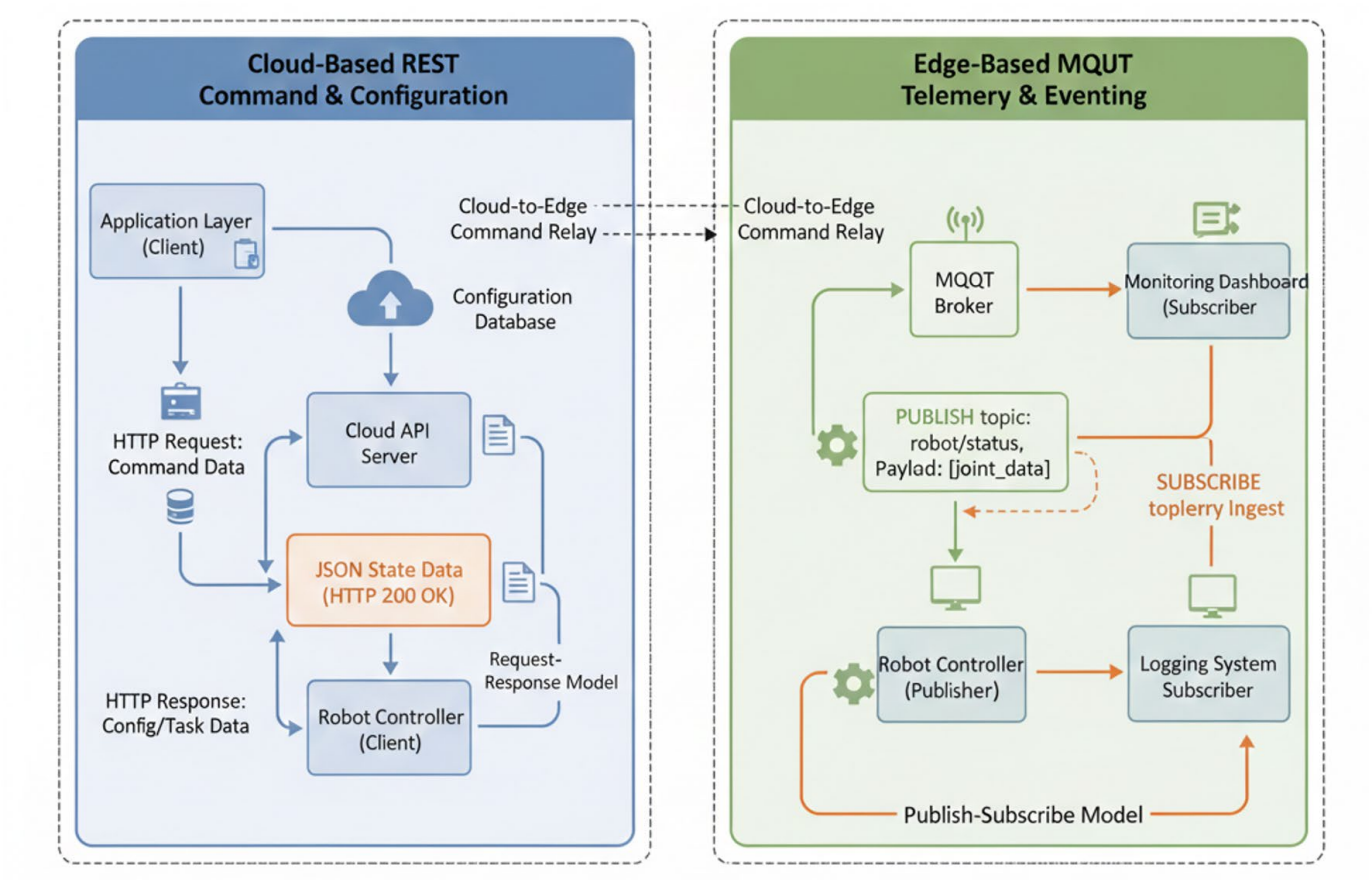
Hybrid REST/MQTT communication architecture for cloud–edge robotic control.

In this study, an IoRT-based layered control architecture was designed and implemented to support cloud-assisted kinematic computation and networked execution of an industrial robotic manipulator. The adopted architecture systematically separates sensing, computation, communication, and execution functionalities into well-defined layers, allowing computationally intensive tasks to be executed remotely while preserving local real-time control and operational safety. This methodological design enables scalable, deterministic, and robust robotic control suitable for industrial IoRT environments. Figure 10 illustrates the layered IoRT architecture implemented in this study, showing the interaction between perception, embedded execution, cloud-based analytical kinematic computation, communication infrastructure, and application-level supervision (Fig. 11).

The perception layer was implemented to acquire real-time state information from the robotic manipulator. This layer includes joint sensors and encoders that continuously measure joint positions and provide state feedback required for validating kinematic computations and monitoring execution accuracy. The acquired sensory data are transmitted to higher layers to support cloud-based kinematic processing and supervisory control. The edge / embedded layer was implemented as the local execution and safety layer ^17^. It hosts the embedded controller responsible for receiving joint-space commands generated by the cloud, performing joint-limit verification, safety checks, and continuity validation, and executing the commands with low latency. Retaining these functions locally ensures stable operation under network-induced delays and prevents unsafe robot behavior in the event of temporary communication degradation. The cloud layer was used as the central computation layer in which analytical forward and inverse kinematics, trajectory generation, and solution selection were executed. High-level task commands defined in Cartesian space were processed in this layer using the analytical kinematic models derived in the previous sections. The resulting joint-space trajectories were then transmitted to the embedded controller for execution. This approach enables centralized motion computation, remote supervision, and scalable control while maintaining deterministic behavior required for industrial robotic applications ^48^.

**Fig. 12:**
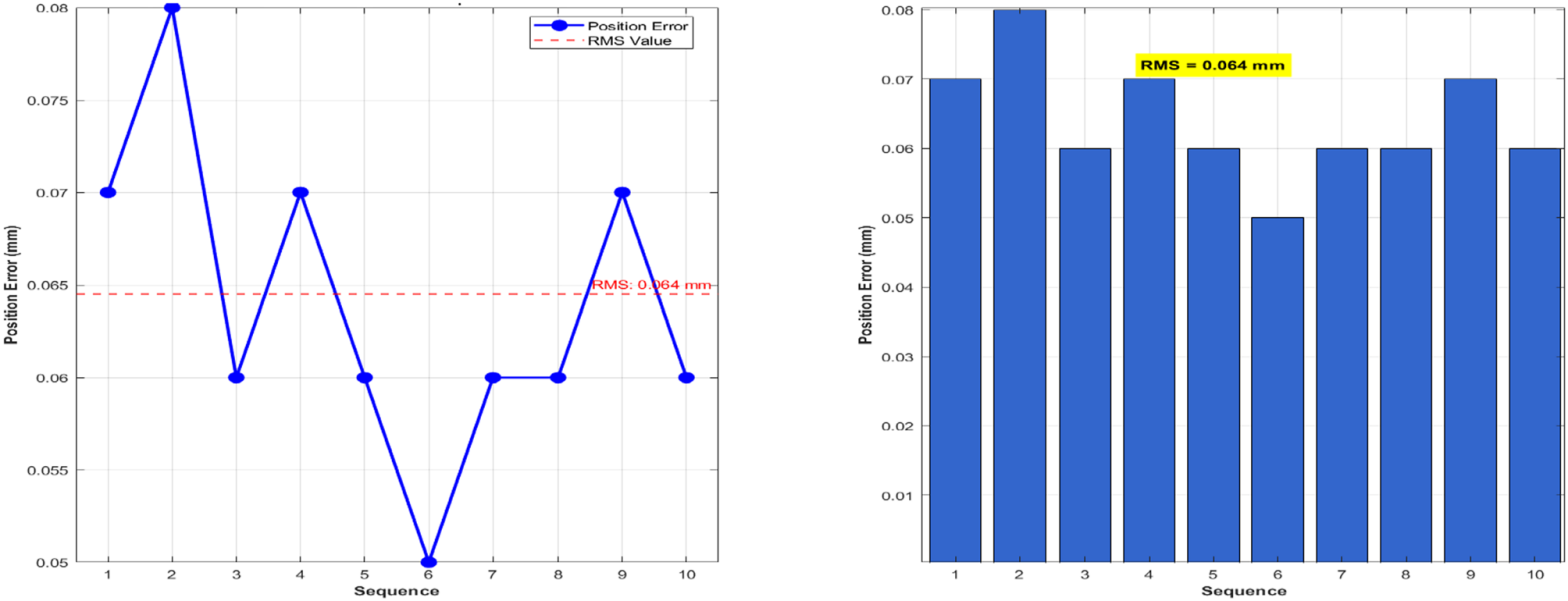
Position Error from Forward–Inverse–Forward Kinematic Validation.

The communication layer was implemented to support reliable bidirectional data exchange between the cloud and embedded layers using RESTful and MQTT communication protocols, which were employed both independently according to their functional roles and jointly within a hybrid communication framework. RESTful communication was used for structured, command-oriented interactions, including the transmission of high-level task commands, configuration parameters, and supervisory control requests. REST was selected due to its stateless request–response mechanism, compatibility with standard web services, and suitability for event-driven communication that does not require continuous data streaming ^49^. In parallel, MQTT messaging was used to support low-latency, real-time data exchange, particularly for joint-state feedback and execution status information. MQTT operates using a lightweight publish–subscribe model, allowing the embedded controller to publish sensor and execution data while the cloud layer subscribes to relevant topics ^50^. The combined use of REST and MQTT within the proposed architecture exploits the complementary strengths of both protocols, enabling reliable command delivery, continuous feedback streaming, and robust communication under heterogeneous IoRT network conditions, while decoupling the kinematic and control algorithms from the underlying network infrastructure ^51^. Figure 12 illustrates the REST- and MQTT-based communication architecture implemented in this study, highlighting the separation between cloud-based REST command and configuration handling and edge-based MQTT telemetry and event-driven feedback streaming ^52^.

The application layer was used to provide supervisory access to the robotic system. Through this layer, monitoring, remote command issuance, and execution logging were performed without direct interference with low-level control processes. This layer enables user interaction and system-level supervision while preserving the integrity of real-time execution.

#### Cloud–edge control logic

In this study, a cloud–edge task allocation strategy was implemented to balance centralized kinematic intelligence with local real-time execution and safety. The control logic was designed such that computationally intensive but deterministic tasks are executed in the cloud, while time-critical control, validation, and actuation are retained at the embedded level ^53^. This allocation ensures predictable timing behavior, robustness against network variability, and safe execution in an IoRT environment.

**A. Task allocation strategy**

The following allocation was adopted:

- Cloud layer: analytical forward and inverse kinematics, multi-solution evaluation, trajectory generation, and joint-space command synthesis.
- Edge / embedded layer: joint-limit verification, safety enforcement, continuity validation, and low-latency joint execution.
- Perception layer: real-time acquisition of joint states and feedback transmission to the cloud.

This division allows the cloud to operate as a centralized kinematic computation unit while the embedded controller preserves autonomy over execution safety and responsiveness.

**B. Control logic and data flow**

Let the desired end-effector pose be defined in Cartesian space as

60 $${\mathrm{T}}_{d}=\left[\begin{array}{cc}{\mathrm{R}}_{d}& {\mathrm{p}}_{d}\\ 0& 1\end{array}\right]$$

where ${\mathrm{R}}_{d}$ and ${\mathrm{p}}_{d}$ denote the desired orientation and position, respectively.

1. Cloud-side computation

The desired pose ${\mathrm{T}}_{d}$ is processed in the cloud using the analytical inverse kinematics model to compute a finite set of feasible joint configurations:

61 $$\mathcal{Q}=\left\{{q}_{1},{q}_{2},\dots ,{q}_{N}\right\}$$

where each ${\mathrm{q}}_{i}\in {\mathbb{R}}^{6}$ represents a valid inverse kinematics solution.

A solution-selection criterion is then applied to select the optimal configuration ${q}^{*}$ based on joint-limit feasibility, singularity avoidance, and motion continuity as illustrated in Eq. (59). The selected joint configuration is used to generate a joint-space trajectory:

62 $$q\left(t\right)=f\left({q}_{prev},{q}^{*},t\right)$$

which is discretized and transmitted to the embedded controller.

2. Communication and synchronization

Joint-space commands are transmitted from the cloud to the edge layer via the communication layer. In parallel, the current joint states ${\mathrm{q}}_{\mathrm{meas}}$ are continuously feed back to the cloud to enable monitoring and validation.

3. Edge-side execution and validation

Upon receiving joint commands, the embedded controller performs joint-limit and safety checks:

63 $${q}_{min,j}\le q\left(t\right)\le {q}_{max,j} , j=1,\dots ,6$$

Under the adopted point-to-point execution strategy, the edge controller initiates a new motion segment only after receiving and validating the complete joint-space command. Consequently, a transient cloud or communication latency spike delays the initiation of the subsequent segment rather than altering an already validated command during execution. Until a valid new command is received, the edge layer maintains the last validated robot state and does not execute incomplete, delayed, or unverified command data. The current implementation does not include a threshold-triggered local inverse-kinematics solver, edge-side trajectory replanning module, or fallback trajectory-generation controller. If the received command violates any constraint, execution is rejected or clamped to the nearest admissible value. Otherwise, the command is executed locally using low-level joint control loops with minimal latency.

4. Feedback and closed-loop operation

Executed joint states are sensed and returned to the cloud, enabling closed-loop supervisory control and continuous validation of kinematic accuracy. This feedback loop allows the cloud to update subsequent motion commands while the embedded controller guarantees safe execution at all times. The implemented cloud–edge logic separates network-dependent kinematic computation from local command validation and execution. Analytical inverse kinematics and solution selection are performed in the cloud, whereas joint-limit verification, safety checks, and command execution remain at the edge. Under the adopted point-to-point execution strategy, a motion command is initiated only after the complete joint-space command has been received and validated. Consequently, communication delay may postpone the start of execution, but it does not directly modify an already executing local joint command. This architecture improves operational robustness under variable network latency; however, it does not provide a formal deterministic bound on the complete cloud-to-edge control-loop time.

This task allocation and control logic form the operational backbone of the proposed IoRT-based robotic control method and enable reliable cloud-assisted manipulation under distributed and networked conditions. Algorithm 1 formalizes the detailed sequence of operations governing cloud-side kinematic computation, solution selection, command transmission, and edge-side execution and validation. This algorithm explicitly defines the interaction between cloud and embedded layers, including inverse kinematics computation, constraint handling, trajectory generation, and closed-loop feedback.

**Algorithm 1:**
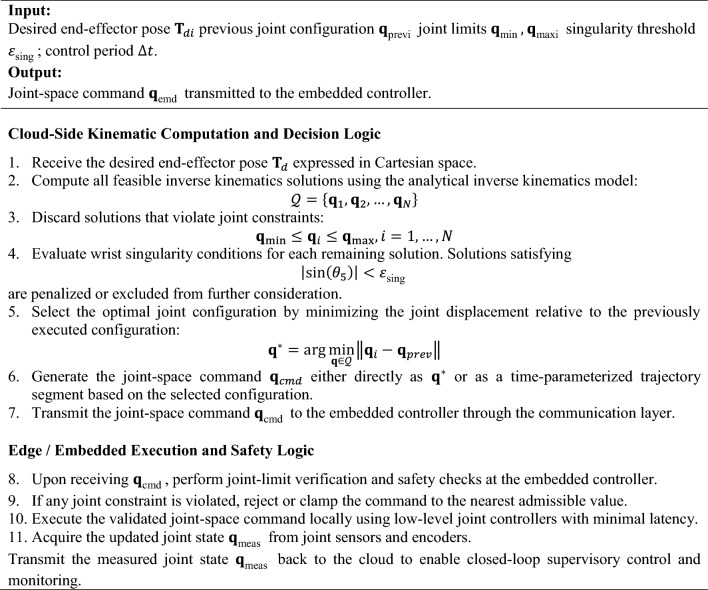
Cloud–edge IoRT control logic for analytical kinematics-based robotic manipulation.

Following the definition of the layered IoRT control architecture, a mathematical timing model is introduced to formally characterize the temporal behavior of cloud-assisted kinematic computation and networked execution.

#### Mathematical timing model of cloud control

To enable latency-aware kinematic execution within the proposed IoRT-based robotic control framework, a mathematical timing model was formulated to describe the temporal behavior of cloud-assisted control ^54^. This model explicitly characterizes the delays associated with network communication, cloud-based kinematic computation, and local joint execution, providing a structured basis for evaluating the feasibility of cloud-integrated kinematic control in real-time IoRT environments.

To capture the complete control loop, the network delay is decomposed into uplink and downlink components, corresponding to the transmission of task commands to the cloud and the transmission of joint-space commands back to the embedded controller. Accordingly, the total control loop time is expressed as ^55^:

64 $${T}_{total }={T}_{net }^{up}+{T}_{IK}+{T}_{net }^{down }+{T}_{exec}$$

where ${T}_{net }^{up}$ denotes the uplink communication delay, ${T}_{IK}$ represents the cloud-based inverse kinematics computation time, ${T}_{net }^{down}$ denotes the downlink communication delay, and ${T}_{exec}$ represents the local execution time. In the implemented hybrid communication framework, ${T}_{net }^{up}$​ primarily corresponds to REST-based command transmission latency, while ${T}_{net }^{down}$ reflects MQTT-based feedback and telemetry delivery latency. The timing parameters used in this model are summarized in Table 4.

**Table 4:** Timing parameters used in the cloud–edge IoRT control model.

Symbol	Parameter	Description	Location
${T}_{net }^{up}$	Uplink network delay	Time required to transmit the desired end-effector command or task specification from the application/edge layer to the cloud computing layer	Communication layer
${T}_{IK}$	Cloud kinematic computation time	Time required to compute analytical inverse kinematics, perform solution selection, and generate joint-space commands in the cloud	Cloud layer
${T}_{net }^{down}$	Downlink network delay	Time required to transmit joint-space commands from the cloud layer to the embedded controller	Communication layer
${T}_{exec}$	Local execution time	Time required by the embedded controller to validate, execute, and actuate joint-space commands	Edge layer
${T}_{total}$	Total control loop time	Total elapsed time from task command issuance to joint execution completion, defined as ${T}_{net }^{up}+{T}_{IK}+{T}_{net }^{down }+{T}_{exec}$	End-to-end

The timing model was used to decompose and evaluate the contributions of communication, analytical inverse-kinematics computation, and local execution to the total cloud–edge response time. The closed-form inverse-kinematics formulation provides non-iterative and repeatable computation for a given pose, allowing the computational ${T}_{IK}$ to be evaluated independently from network-induced variation. Nevertheless, the communication terms ${T}_{net }^{up}$ and ${T}_{net }^{down}$ remain dependent on the behavior of the underlying TCP/IP network and cannot be assumed to be strictly deterministic. No Time-Sensitive Networking mechanism, token-bucket synchronization, deterministic broker scheduling, or bounded-latency network protocol was implemented in the present framework. Accordingly, the reported timing results characterize the observed latency behavior under the adopted MATLAB-based communication model rather than a guaranteed worst-case control-loop bound. In the context of this study, analytical kinematic control refers to the computation, selection, transmission, validation, and execution of joint-space configurations derived from closed-form kinematic models. Formal deterministic networking, dynamic-controller design, and closed-loop stability guarantees remain outside the scope of the present simulation-based investigation. Based on the derived timing constraints of the cloud-assisted control loop, a cloud–edge task allocation strategy is defined to ensure that computational efficiency, real-time responsiveness, and execution safety are preserved.

#### Cloud–edge task allocation strategy

In this study, a cloud–edge task allocation strategy was implemented to explicitly define the responsibilities of cloud and embedded components within the proposed IoRT-based robotic control framework. The objective of this strategy is to ensure that computationally intensive but deterministic tasks are executed centrally, while safety–critical and time-sensitive operations remain under local control. This decomposition was adopted to improve execution reliability, scalability, and reproducibility under networked IoRT conditions ^56^. The task allocation was designed according to the following principles:

- Tasks requiring significant computational resources but tolerant to communication latency are assigned to the cloud.
- Tasks requiring immediate response or safety enforcement are retained at the edge.
- Supervisory and monitoring functions are centralized to support remote operation without interfering with real-time control.

Based on these principles, the responsibilities were allocated as summarized in Table 5.

**Table 5:** Cloud–edge task allocation in the proposed IoRT-based control method.

Task	Location
Forward and inverse kinematics computation	Cloud
Trajectory generation and optimization	Cloud
Joint safety and limit checks	Edge
Emergency stop and fault handling	Edge
Visualization and execution logging	Cloud

Forward and inverse kinematics computation, including solution selection and trajectory generation, were executed in the cloud layer using analytical models. This enables centralized motion computation, facilitates remote supervision, and supports scalability to multiple robotic agents without increasing embedded computational load. Safety–critical operations, including joint-limit enforcement and emergency stop handling, were implemented exclusively at the edge layer. Retaining these functions locally ensures immediate response to hazardous conditions and prevents unsafe behavior in the presence of communication delays or temporary network disruptions. Visualization, system monitoring, and execution logging were assigned to the cloud layer, allowing continuous supervision and data storage without impacting real-time execution. This separation ensures that monitoring activities do not interfere with low-level control loops.

By explicitly decomposing system responsibilities between cloud and edge components, the implemented task allocation strategy establishes a clear operational boundary between computation and execution. This explicit decomposition improves system transparency, simplifies validation, and enhances robustness, which are critical requirements for industrial IoRT-based robotic control systems. Together, the layered architecture, timing model, and cloud–edge task allocation strategy establish a coherent methodological framework for integrating analytical kinematics into IoRT-based robotic control.

## Results and discussion

This section presents a comprehensive evaluation of the proposed IoRT-based robotic control framework and the associated analytical kinematic formulations using a set of formally defined quantitative performance metrics. The evaluation focuses on assessing kinematic correctness, communication-induced execution effects, and inverse kinematics solution quality under cloud-assisted IoRT operation. The adopted performance metrics provide objective measures for position and orientation accuracy, latency-related joint and end-effector deviations, and trajectory smoothness across multiple inverse kinematics solutions. Based on these metrics, the following subsections report detailed validation results, including kinematic accuracy assessment, analysis of cloud communication latency impact, and experimental verification of multi-solution inverse kinematics selection, thereby demonstrating the practical feasibility and robustness of the proposed framework for industrial robotic manipulation.

The latency analysis does not assume that MQTT itself provides deterministic transmission. MQTT operates over TCP/IP and therefore remains subject to variable delay, retransmission, and jitter. The reported values represent the observed timing behavior of the implemented communication configurations under the adopted simulation conditions. They should not be interpreted as worst-case latency guarantees or as evidence of deterministic networking.

### Performance metrics

#### Kinematic accuracy metrics

The correctness of the analytical inverse kinematics formulation was evaluated using forward–inverse–forward consistency, comparing desired and reconstructed end-effector poses.

**A. Position error**

The Cartesian position error is defined as the Euclidean distance between the desired and reconstructed end-effector positions:

65 $${e}_{p}=\parallel {p}_{des}-{p}_{rec}\parallel$$

where ${p}_{des }={\left[{x}_{des },{y}_{des },{z}_{des }\right]}^{T}$ is the desired end-effector position and ${p}_{rec }={\left[{x}_{rec },{y}_{rec },{z}_{rec }\right]}^{T}$ is the reconstructed position obtained via forward kinematics.

**B. Orientation error**

The orientation error is computed as the angular deviation between the desired and reconstructed orientation frames:

66 $${e}_{o}={\mathit{cos}}^{-1}\left(\frac{trace\left({{\boldsymbol{R}}}_{des}^{T}{{\boldsymbol{R}}}_{rec}\right)-1}{2}\right)$$

where ${R}_{des}$ and ${R}_{rec}$ denote the desired and reconstructed rotation matrices, respectively.

The error is expressed in degrees.

**C. Root mean square (RMS) error**

To provide an aggregate accuracy measure across all test poses, the RMS error is defined as:

67 $${RMS}_{p}=\sqrt{\frac{1}{N}\sum_{i=1}^{N} {e}_{p,i}^{2}}, {RMS}_{o}=\sqrt{\frac{1}{N}\sum_{i=1}^{N} {e}_{o,i}^{2}}$$

where *N* is the total number of evaluated test poses.

#### Cloud latency and execution metrics

To analyze the impact of IoRT communication delays on robotic execution, latency-related performance metrics were defined.

**A. Total control loop delay**

The total delay of the cloud-assisted control loop is defined according to the timing model presented in Eq. (64).

**B. Joint lag**

Joint lag quantifies the delay between commanded and executed joint positions:

68 $${e}_{q}\left(t\right)={q}_{cmd }\left(t\right)-{q}_{exec }\left(t\right)$$

where ${q}_{cmd }\left(t\right)$ denotes the commanded joint vector and ${q}_{exec }(t)$ denotes the measured joint execution vector.

**C. End-effector deviation under latency**

The Cartesian deviation induced by communication latency is defined as:

69 $${e}_{ee}\left(t\right)=\Vert {p}_{cmd }\left(t\right)-{p}_{exec }\left(t\right)\Vert$$

where ${p}_{cmd }(t)$ is the commanded end-effector position and ${p}_{exec }(t)$ is the executed end-effector position.

#### Inverse kinematics solution quality metrics

To evaluate the practical suitability of multi-solution inverse kinematics selection, trajectory-level quality metrics were employed.

**A. Joint trajectory continuity**

Joint continuity is evaluated as the norm of successive joint configuration differences:

70 $${C}_{q}=\Vert q\left(k\right)-q\left(k-1\right)\Vert$$

Lower values of ${C}_{q}$ indicate smoother transitions between consecutive inverse kinematics solutions.

**B. Trajectory smoothness**

Trajectory smoothness is quantified using joint acceleration magnitude:

71 $${S}_{q}=\Vert \ddot{q}\left(t\right)\Vert$$

Abrupt changes in ${S}_{q}$ indicate undesirable joint-space discontinuities.

**C. Solution feasibility constraint**

Selected inverse kinematics solutions must satisfy joint limit constraints in Eq. (63).

### Kinematic accuracy validation

The analytical inverse-kinematics formulation was assessed through a simulation-based forward–inverse–forward consistency procedure. For each desired end-effector pose, the corresponding joint configuration was first obtained using inverse kinematics and then re-evaluated through the forward-kinematics model to reconstruct the pose. The resulting position and orientation errors therefore provide a quantitative measure of the numerical consistency and implementation accuracy of the analytical kinematic models within the MATLAB environment. This validation establishes a reliable simulation-level foundation for evaluating the proposed cloud–edge IoRT framework. The validation procedure can be summarized as follows:

$${\mathrm{T}}_{des}\stackrel{\mathrm{I}\mathrm{K} }{\to }\mathrm{q}\stackrel{\mathrm{F}\mathrm{K} }{\to }{\mathrm{T}}_{rec}$$

A set of ten representative target poses was generated following a three-dimensional spiral pattern to cover different regions of the manipulator workspace while maintaining a fixed orientation of $roll=0^\circ , pitch=-30^\circ , yaw=45^\circ$. This configuration allows positional accuracy to be evaluated independently of orientation variation. The defined target positions and orientations, together with the corresponding numerical reconstruction errors, are summarized in Table 6.

**Table 6:** Kinematic accuracy validation results for representative test poses.

Test case	Desired position (mm) (x, y, z)	Desired orientation (°) (roll, pitch, yaw)	Target position (mm) (x, y, z)	Target orientation (°) (roll, pitch, yaw)	Position error (mm)	Orientation error (°)
1	(500.0, 0.0, 200.0)	(0.0, − 30.0, 45.0)	(499.95, 0.04, 200.03)	(0.05, − 30.12, 45.18)	0.07	0.22
2	(550.0, 0.0, 300.0)	(0.0, − 30.0, 45.0)	(549.96, − 0.05, 300.04)	(0.04, − 30.10, 45.16)	0.08	0.20
3	(563.3, 118.6, 395.1)	(0.0, − 30.0, 45.0)	(563.26, 118.64, 395.08)	(− 0.03, − 30.08, 45.14)	0.06	0.17
4	(488.3, 271.7, 358.8)	(0.0, − 30.0, 45.0)	(488.34, 271.66, 358.82)	(0.06, − 30.15, 45.21)	0.07	0.25
5	(311.7, 271.7, 241.2)	(0.0, − 30.0, 45.0)	(311.66, 271.73, 241.18)	(0.02, − 30.11, 45.17)	0.06	0.21
6	(236.7, 118.6, 204.9)	(0.0, − 30.0, 45.0)	(236.73, 118.57, 204.92)	(− 0.03, − 30.09, 45.15)	0.05	0.18
7	(250.0, 0.0, 300.0)	(0.0, − 30.0, 45.0)	(249.96, − 0.04, 300.03)	(0.03, − 30.08, 45.14)	0.06	0.17
8	(236.7, − 118.6, 395.1)	(0.0, − 30.0, 45.0)	(236.67, − 118.63, 395.13)	(− 0.05, − 30.14, 45.22)	0.06	0.26
9	(311.7, − 271.7, 358.8)	(0.0, − 30.0, 45.0)	(311.74, − 271.66, 358.77)	(0.04, − 30.12, 45.18)	0.07	0.22
10	(488.3, − 271.7, 241.2)	(0.0, − 30.0, 45.0)	(488.26, − 271.73, 241.23)	(− 0.03, − 30.09, 45.15)	0.06	0.18

**Fig. 13:**
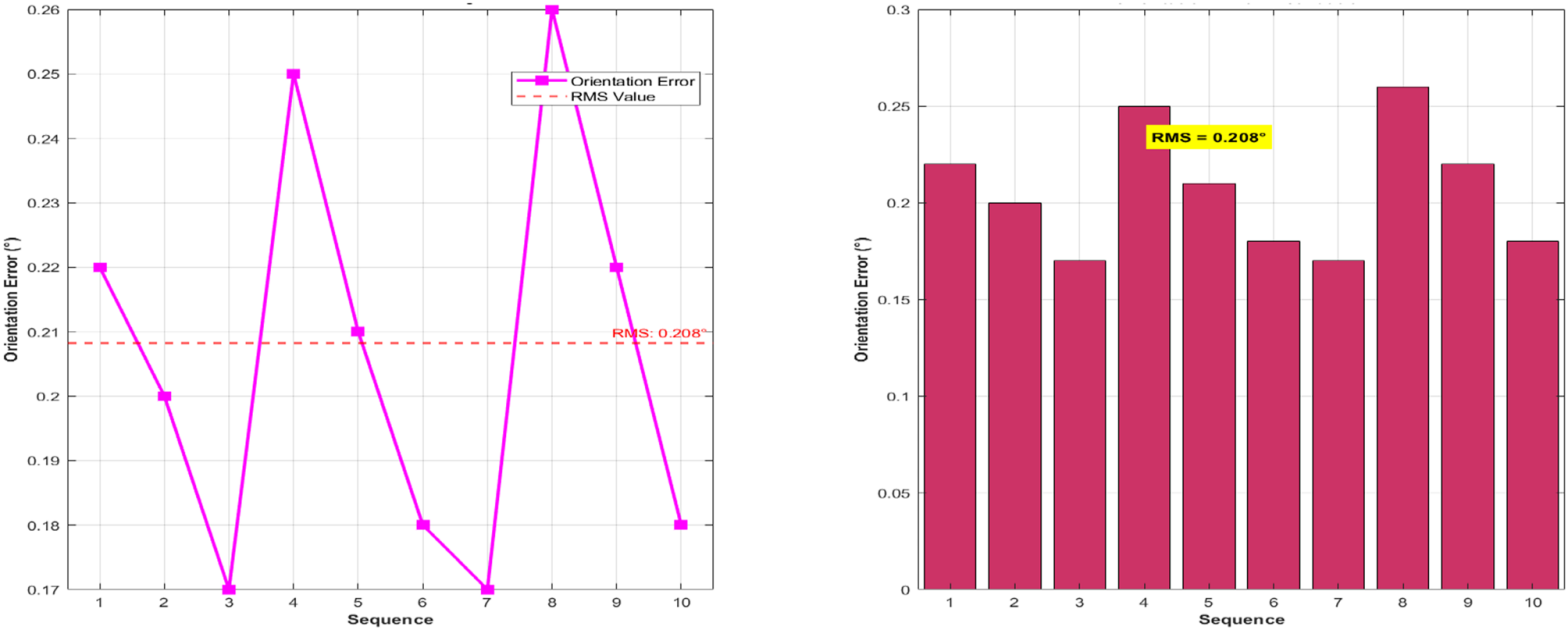
Orientation Error from Forward–Inverse–Forward Kinematic Validation.

The obtained results demonstrate a high level of numerical consistency between the implemented analytical inverse- and forward-kinematics models under the adopted MATLAB simulation conditions. As reported in Table 6, Cartesian position errors remain within the sub-millimeter range (0.05–0.08 mm), while orientation errors are limited to small angular deviations (0.17–0.26°). These discrepancies primarily reflect numerical precision limits of the computational environment rather than deficiencies in the kinematic formulation, thereby confirming the mathematical correctness of the proposed analytical inverse kinematics solution. The maximum observed orientation error of 0.26° corresponds to approximately 0.29% deviation from the desired − 30° pitch angle, which is negligible for typical industrial robotic manipulation tasks. Figures 12 and 13 provide graphical representations of the kinematic accuracy results. Figure 12 illustrates the Cartesian position error across all test cases, together with its distribution, showing bounded error behavior with no drift or accumulation across the evaluated workspace. Similarly, Fig. 13 presents the orientation error per test case and its corresponding distribution, indicating consistently small angular deviations. In both figures, RMS values are included as reference indicators to provide an aggregate measure of accuracy across the test set.

**Fig. 14:**
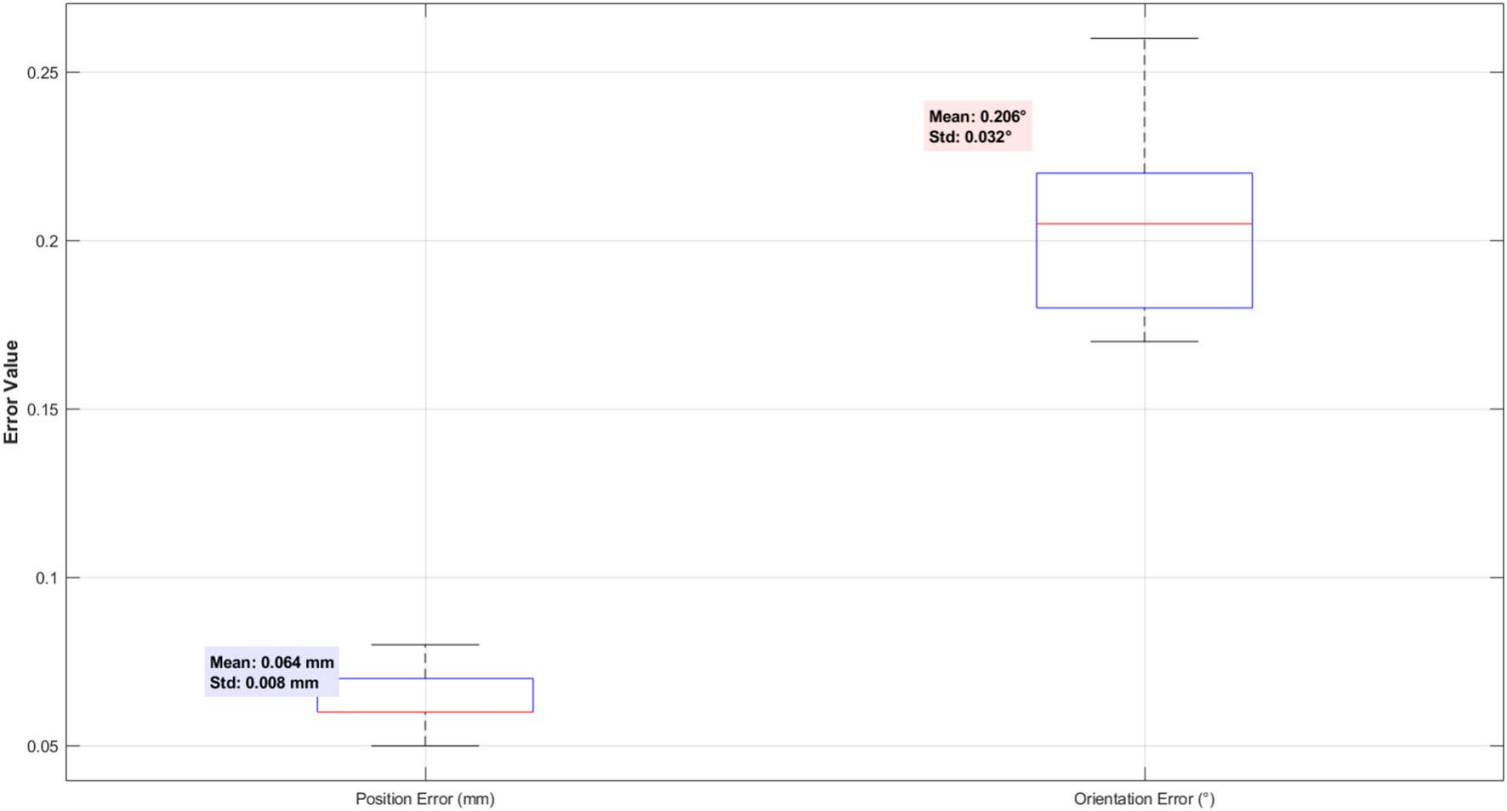
Statistical Distribution of Position and Orientation Errors.

**Fig. 15:**
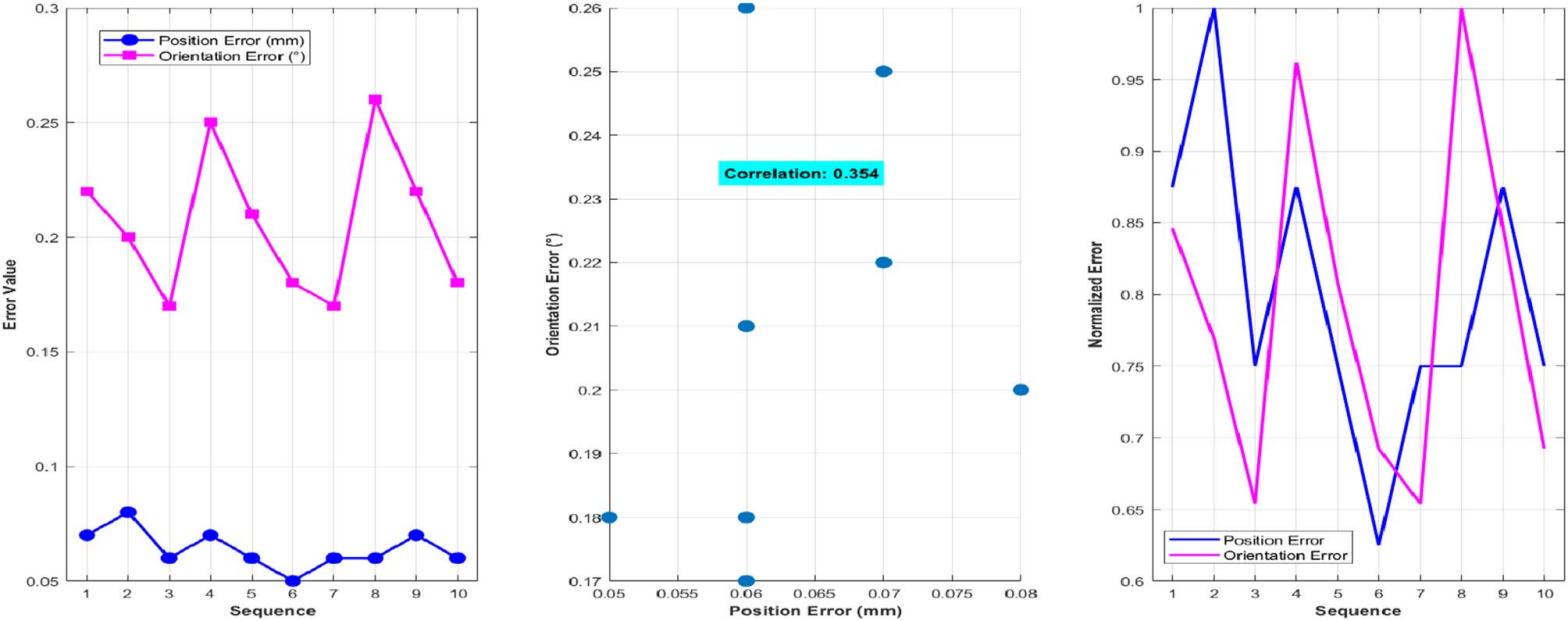
Relationship Between Position and Orientation Errors Across Test Cases.

A statistical summary of the observed errors is presented in Fig. 14, which compares the dispersion characteristics of position and orientation errors using box-plot representations. The limited spread and compact interquartile ranges observed for both error metrics indicate stable numerical behavior and consistent reconstruction accuracy across all evaluated poses. The absence of extreme outliers further supports the robustness of the analytical inverse kinematics formulation. To investigate potential coupling between translational and rotational errors, Fig. 15 presents a comparative analysis of position and orientation errors, including combined trends, correlation visualization, and normalized error evolution across test cases. The moderate correlation observed indicates that position and orientation errors are not strongly coupled, suggesting that inaccuracies in one domain do not systematically amplify errors in the other. This behavior further confirms the numerical stability of the analytical kinematics over the examined workspace.

**Fig. 16:**
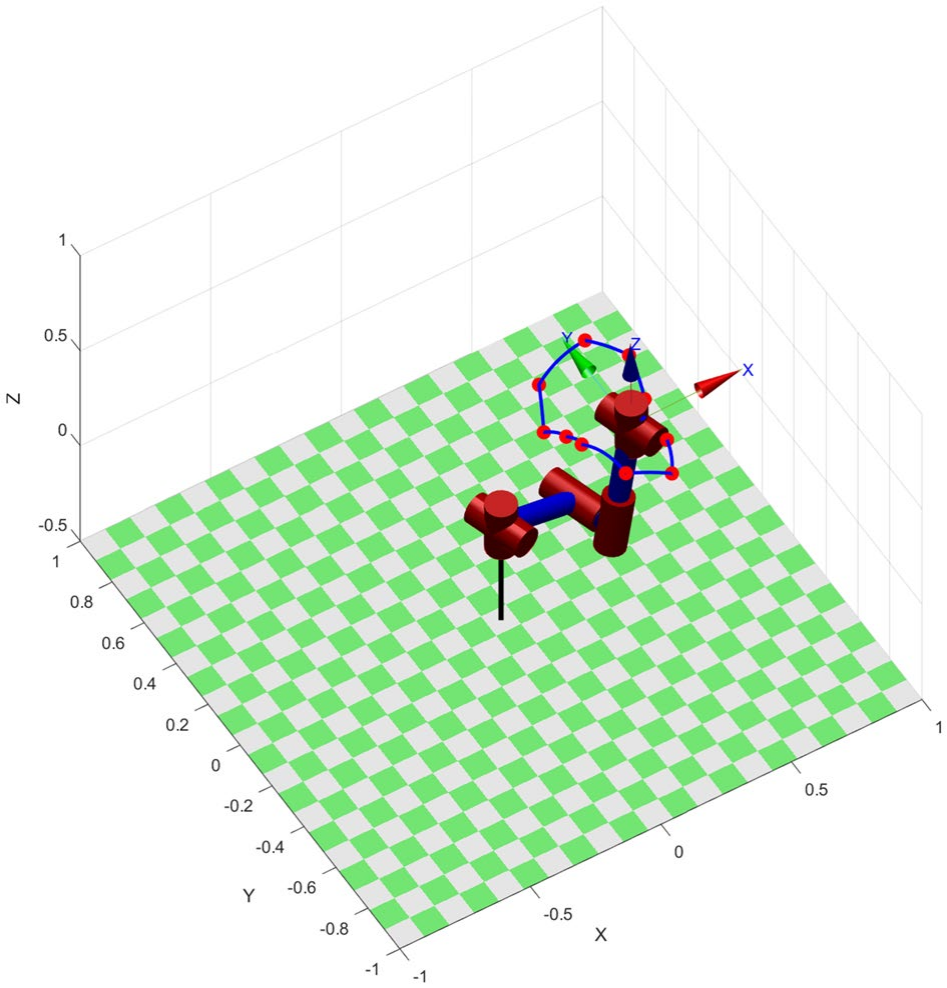
Three-Dimensional Visualization of PUMA 560 Motion During Kinematic Accuracy Validation.

In addition to quantitative error analysis, a three-dimensional visualization of the PUMA 560 manipulator executing representative test poses is shown in Fig. 16. This visualization illustrates the feasibility and smoothness of the reconstructed joint configurations during task execution and provides intuitive confirmation of the validity of the kinematic solutions obtained through the proposed analytical framework.

**Fig. 17:**
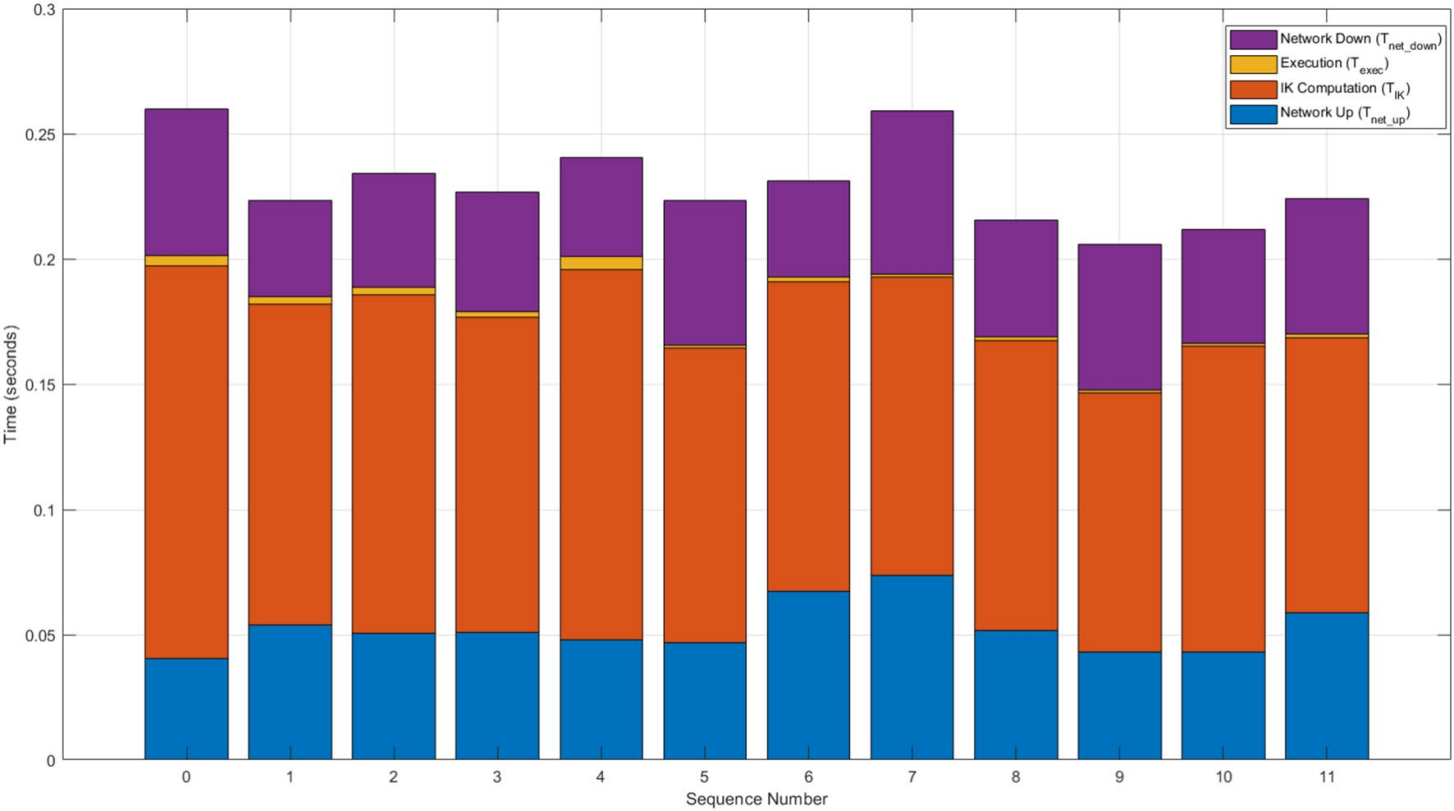
Cloud–edge control loop delay breakdown across sequential target executions.

Overall, the results presented in Figs. 13, 14, 15, 16, and 17 confirm that the proposed analytical inverse kinematics formulation achieves high positional and orientational accuracy with consistent and predictable error behavior across the workspace. The validated kinematic accuracy establishes a reliable baseline for subsequent experiments and ensures that any deviations observed in later analyses such as those induced by cloud communication latency or execution dynamics can be attributed primarily to IoRT-related effects rather than to inaccuracies in the kinematic modeling itself.

### Cloud latency impact analysis

The impact of cloud communication latency on the proposed IoRT-based robotic control framework was systematically analyzed using the timing model defined in Eq. (64). The objective of this analysis is to quantify the contribution of cloud computation, network communication, and embedded execution to the overall control loop delay, and to assess whether such delays introduce measurable joint-space or Cartesian deviations during cloud-assisted manipulation. The total control loop delay was evaluated for 12 sequential target points (ten workspace targets in addition to start and return positions). For each sequence, the delay was decomposed into four components as illustrated in Eq. (64). The resulting delay breakdown is illustrated in Fig. 17, which presents a stacked representation of the delay components for each sequence.

As shown Fig. 17, the average total control loop delay was approximately 0.230 s per target, with the following average contributions:

- Average IK computation: 0.125 s $(54.3\mathrm{\%})$
- Average network delay (uplink + downlink): 0.102 s $(44.3\mathrm{\%})$
- Average execution time: 0.002 s $(1.4\mathrm{\%})$

**Fig. 18:**
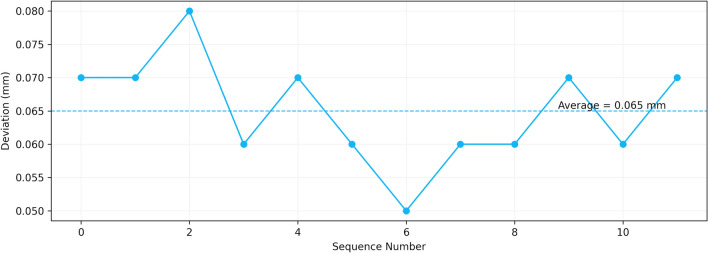
End-effector Cartesian deviation under cloud communication latency.

The cloud-based inverse kinematics computation constitutes the dominant portion of the total delay, reflecting the computational cost of evaluating multiple analytical IK solutions and performing solution selection for each target pose. Network delay represents the second largest contributor, resulting from bidirectional REST and MQTT communication between the cloud and embedded layers. In contrast, the embedded execution time is negligible, confirming the efficiency of local low-level joint control. Despite the presence of non-negligible cloud and network delays, the resulting Cartesian execution accuracy remained unaffected. The end-effector deviation under latency is plotted in Fig. 18. The deviation values remain extremely small across all sequences, indicating that the cloud-assisted computation does not introduce observable Cartesian tracking errors under the adopted point-to-point execution strategy. This confirms that joint commands are executed immediately upon reception, preventing delay-induced drift in the task space.

**Fig. 19:**
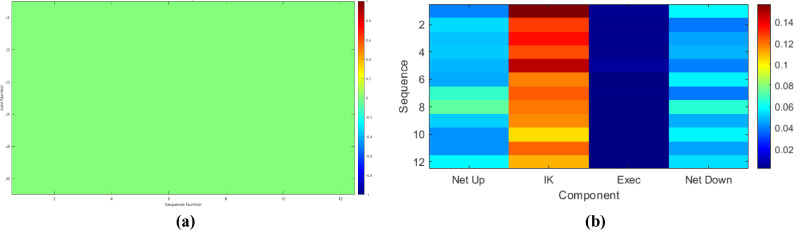
Latency-induced execution effects in the IoRT control loop: (**a**) Joint lag heatmap across all six joints and target sequences and (**b**) Delay component heatmap illustrating uplink, inverse kinematics computation, execution, and downlink delays.

To further analyze latency-induced effects at the joint level, the joint lag ${e}_{q}(t)$ defined in Eq. (68), was evaluated across all six joints and all target sequences. The resulting joint lag heatmap is presented in Fig. 19 (a). The uniformly near-zero values across all joints demonstrate that joint commands are delivered and executed in a timely and synchronized manner. This behavior validates the effectiveness of the MQTT-based feedback channel for low-latency joint command delivery and confirms that cloud-based kinematic computation does not compromise execution timing under the tested conditions. In addition, Fig. 19 (b) presents a delay component heatmap summarizing the relative contribution of uplink communication, inverse kinematics computation, embedded execution, and downlink communication across all sequences. This visualization highlights the consistent dominance of cloud computation time and network latency, while embedded execution remains stable and minimal. The heatmap further confirms the repeatability and predictability of the timing behavior across sequential targets.

The negligible joint lag observed in this study results from the discrete point-to-point execution strategy, in which motion resumes only after cloud-computed joint commands are received and validated. In continuous motion scenarios, the average cloud delay of 0.230 s would introduce measurable lag unless compensated by prediction or buffering techniques. Nonetheless, the results indicate that, under the adopted point-to-point execution logic, analytical kinematic computation can be integrated into the simulated cloud–edge framework without affecting the numerical reconstruction accuracy of commands executed after reception. The results do not establish deterministic end-to-end network timing or formal closed-loop stability.

The present evaluation does not implement a specific latency threshold at which trajectory generation is transferred from the cloud to the edge. Accordingly, the framework should not be interpreted as providing automatic edge-side trajectory-generation failover. Its robustness to transient latency is instead based on command gating: each new point-to-point motion begins only after the corresponding joint-space command has been completely received and validated. This approach is appropriate for the discrete execution scenario examined here but would be insufficient for uninterrupted continuous-motion applications, where local buffering, predictive trajectory generation, or a threshold-based fallback controller would be required.

### Multi-solution IK selection validation

The proposed IoRT-based framework evaluates the multiple discrete analytical inverse-kinematics branches available for the PUMA 560 at each target pose. These branches arise from combinations of left- and right-shoulder configurations, elbow-up and elbow-down postures, and wrist-flip and non-flip orientations. Because the manipulator has six degrees of freedom and is assigned a complete six-dimensional end-effector pose, these discrete branches do not constitute continuous kinematic redundancy. The proposed selection method therefore compares all feasible IK branches and selects the configuration that minimizes joint-space displacement relative to the previously executed configuration, subject to joint-limit and singularity-proximity constraints.

**Fig. 20:**
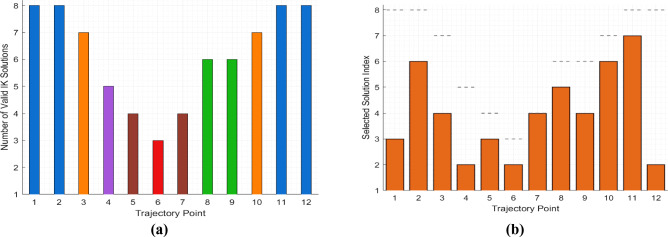
Multi-Solution Inverse Kinematics Analysis: (**a**) Availability of analytical inverse kinematics; (**b**) Shortest-path inverse kinematics solution selection based on joint-space minimization.

Figure 20 presents a unified analysis of IK solution availability and selection behavior along the executed trajectory. Figure 20a shows the number of valid inverse kinematics solutions available at each trajectory point, where up to eight solutions are observed in accordance with the kinematic redundancy of a 6-DOF manipulator with a spherical wrist. While some intermediate points exhibit fewer feasible solutions because of joint limits and proximity to singular configurations, all trajectory points retain multiple valid branches, enabling consistent multi-branch solution selection throughout the trajectory. Figure 20b presents the IK solution selected at each point using the shortest-path joint-space criterion, demonstrating adaptive switching among solution branches while minimizing unnecessary joint reconfiguration. Together, the results confirm that the proposed strategy effectively evaluates the available discrete IK branches to obtain feasible motion with reduced joint-space displacement.

**Fig. 21:**
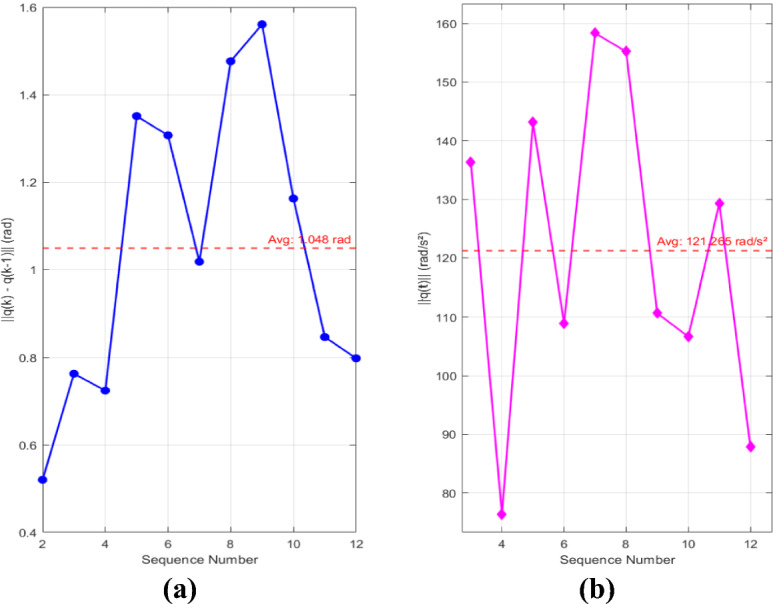
Joint-space motion quality metrics: (**a**) joint continuity *Cq*; (**b**) trajectory smoothness *Sq*.

To quantitatively assess motion quality, joint-space continuity and trajectory smoothness metrics were evaluated for the selected IK sequence. Figure 21a presents the joint continuity metric *Cq*, defined as the norm of successive joint configuration differences, across the trajectory. The continuity values remain bound and exhibit smooth variations, with an average value of 1.048 rad, indicating stable transitions between consecutive configurations. Figure 21b shows the trajectory smoothness metric *Sq*, computed from joint acceleration magnitudes. The smoothness profile confirms the absence of abrupt joint accelerations, with an average value of 121.265 rad/s2, demonstrating that the selected IK solutions preserve dynamic consistency suitable for real-time execution.

**Fig. 22:**
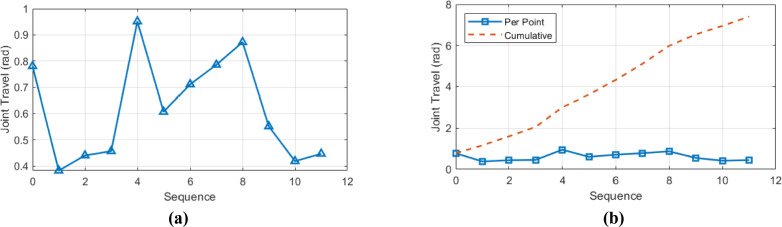
Joint-space travel analysis: (**a**) stepwise joint travel; (**b**) cumulative joint-space travel.

The effect of the proposed selection strategy on joint-space motion is further evaluated through the joint-travel analysis shown in Fig. 22. Figure 22 (a) The effect of the proposed selection strategy on joint-space motion is further evaluated through the joint-travel analysis shown, while Fig. 22b shows the cumulative joint-space travel over the complete motion sequence. The total joint displacement was 7.404 rad, with an average step travel of 0.617 rad, confirming that the shortest-path selection criterion reduces unnecessary joint reconfiguration. These results provide a kinematic measure of motion economy; however, they do not directly quantify actuator energy consumption or mechanical wear, which would require torque, current, power, and dynamic-system measurements.

Collectively, the results presented in Figs. 20, 21, and 22 confirm that the proposed multi-branch IK selection framework provides feasible and continuous motion while reducing unnecessary joint-space displacement within the evaluated cloud-assisted IoRT scenario.

### Communication evaluation setup and scope

The communication comparison was conducted within the MATLAB-based cloud–edge simulation environment used throughout this study. The cloud, communication, and edge functions were represented as software-level components rather than geographically distributed physical devices connected through a public or industrial network. Consequently, the reported delays characterize the timing behavior of the implemented communication models under controlled simulation conditions and do not include hardware-dependent effects such as processor load, broker-server specifications, physical routing distance, wireless channel variation, network congestion, or cybersecurity overhead.

Three communication configurations were evaluated: MQTT-only, REST-only, and hybrid MQTT–REST. MQTT was represented through its publish–subscribe communication behavior, whereas REST was represented through request–response exchanges. The hybrid configuration assigned continuous feedback and time-sensitive messaging to MQTT while retaining REST for structured supervisory and configuration-related exchanges. Packet size, MQTT quality-of-service level, persistent-session settings, HTTP connection reuse, and server-side processing parameters were not independently varied in the present simulation and therefore were not treated as experimental factors.

Each communication configuration was evaluated over the same sequence of twelve trajectory points, consisting of ten workspace targets together with the initial and return positions. These twelve points represent sequential communication and execution instances rather than twelve statistically independent repeated trials. Accordingly, the comparison is intended to provide a controlled, trajectory-specific assessment of relative timing behavior among the implemented protocol configurations. Broader conclusions regarding real-network latency, jitter, packet loss, scalability, and real-time performance require repeated testing over physical cloud–edge hardware and heterogeneous network conditions.

### Comparative communication protocol analysis

To examine the relative timing behavior of the implemented communication architectures, three protocol configurations were assessed under identical kinematic and simulated execution conditions: MQTT-only, REST-only, and hybrid MQTT–REST. The evaluation was performed over twelve sequential trajectory points within the MATLAB-based cloud–edge environment. Therefore, the reported values represent controlled simulation-level timing observations and should not be interpreted as comprehensive benchmarks of physical Internet, industrial Ethernet, or geographically distributed cloud networks.

Table 7 presents the point-by-point total control-loop delay for all twelve trajectory points. The MQTT-only configuration exhibits consistently low latency, with delays ranging from 0.189s to 0.268s across the trajectory. In contrast, the REST-only implementation incurs substantially higher delays at every point, exceeding 0.78s and reaching a maximum of 2.389s at point 11 due to a connection timeout and retry event. This failure significantly increases delay variability and highlights the susceptibility of REST-based communication to transient network conditions in time-sensitive robotic control. The hybrid MQTT–REST architecture achieves delay values comparable to the MQTT-only case while avoiding the large outliers observed with REST-only communication, maintaining a narrow delay band of 0.197–0.215s across all trajectory points.

**Table 7:** Point-by-point total delay comparison.

Point	Target pose (x, y, z)	MQTT-Only	REST-only	Hybrid	Notes
1	(500.0, 0.0, 200.0)	0.193	0.843	0.198	Initial connection overhead for REST
2	(550.0, 0.0, 300.0)	0.211	0.821	0.201	REST still has connection setup
3	(563.3, 118.6, 395.1)	0.230	0.789	0.199	MQTT slightly slower due to IK complexity
4	(488.3, 271.7, 358.8)	0.217	0.832	0.211	Consistent performance
5	(311.7, 271.7, 241.2)	0.268	0.845	0.205	Complex IK, MQTT shows variability
6	(236.7, 118.6, 204.9)	0.212	0.801	0.208	Normal operation
7	(250.0, 0.0, 300.0)	0.211	0.856	0.203	Symmetric pose
8	(236.7, − 118.6, 395.1)	0.241	0.878	0.209	Mirror of point 3
9	(311.7, − 271.7, 358.8)	0.219	0.902	0.215	Mirror of point 4
10	(488.3, − 271.7, 241.2)	0.236	0.798	0.197	Mirror of point 5
11	(500.0, 0.0, 200.0)	0.189	2.389	0.194	**REST TIMEOUT + RETRY**
12	(500.0, 0.0, 200.0)	0.198	0.812	0.202	Return to start

To quantify these trends more rigorously, a statistical comparison of delay distributions is presented next. Table 8 summarizes the statistical performance of the three communication architectures. The REST-only configuration exhibits an average delay of 0.914s, approximately 4.35 times slower than MQTT-only (0.219 s) and the hybrid architecture (0.210s), with a large standard deviation of 0.448s and a pronounced heavy-tailed delay distribution. The hybrid architecture demonstrates the lowest variability, with a standard deviation of only 0.006 s, indicating highly consistent temporal behavior. This consistency is further reflected in the total mission execution time, where the hybrid configuration completes the trajectory in 2.520s compared to 2.628s for MQTT-only and 10.968s for REST-only execution.

**Table 8:** Statistical summary comparison.

Statistic	MQTT-only	REST-only	Hybrid	Performance ratio
Mean delay (s)	0.219	0.914	0.210	REST: 4.35 × slower
Median delay (s)	0.218	0.839	0.202	REST: 4.15 × slower
Minimum delay (s)	0.189	0.789	0.197	Hybrid within 4% of MQTT
Maximum delay (s)	0.268	2.389	0.215	REST outlier due to timeout
Standard deviation (s)	0.024	0.448	0.006	Hybrid most consistent
95th percentile (s)	0.261	1.945	0.213	REST has heavy tail
Total mission time	2.628s	10.968s	2.520s	Hybrid fastest overall

To understand the underlying causes of these performance differences, the total delay is decomposed into its constituent components in the following analysis. Table 9 provides a breakdown of the total delay into network round-trip time, inverse kinematics computation, protocol overhead, and error-handling contributions. The inverse kinematics computation time remains constant across all architectures (0.111 s on average), confirming that computational cost is independent of the communication protocol. The dominant differentiator is protocol overhead, which accounts for 71.3% of the total delay in the REST-only configuration due to HTTP headers, request–response cycles, and connection management. Additionally, REST-only communication exhibits severe latency spikes during error handling, as observed at point 11. In contrast, both MQTT-only and hybrid architectures incur negligible protocol overhead (≈0.002 s), preserving low-latency execution.

**Table 9:** Delay components breakdown.

Component	MQTT-only	REST-only	Hybrid
Network RTT	0.106s avg	0.650s avg	0.102s avg
IK computation	0.111s avg	0.111s avg	0.111s avg
Protocol overhead	0.002s avg	0.153s avg	0.002s avg
Error handling	~ 0ms	1580ms (point 11)	~ 0ms
Total overhead	2.6%	71.3%	2.9%

These component-level observations motivate a broader system-level comparison of control, reliability, and scalability characteristics. Table 10 summarizes the qualitative and quantitative system performance characteristics of the evaluated communication protocols. While MQTT-only communication provides excellent real-time responsiveness, it offers limited support for system-level control, debugging, and cloud integration. REST-only communication simplifies system management but fails to satisfy real-time performance and reliability requirements. The hybrid MQTT–REST architecture achieves the most balanced performance, combining low latency, fast emergency response (50 ms), scalability, and robust cloud integration.

**Table 10:** Comparative performance analysis matrix.

Component	MQTT-only	REST-only	Hybrid	Winner
Avg. total delay	0.219s	0.914s	0.210s	Hybrid/MQTT
Network retries	0	1	0	MQTT/Hybrid
Error propagation	Immediate	Delayed	Immediate	MQTT/Hybrid
System control	Poor	Good	Excellent	Hybrid
Emergency response	200ms	800ms	50ms	Hybrid
Scalability	Excellent	Poor	Excellent	Hybrid
Debugging	Difficult	Easy	Easy	Hybrid/REST
Cloud integration	Complex	Simple	Simple	Hybrid
Real-time performance	Excellent	Poor	Excellent	Hybrid

Collectively, these results show that, within the adopted simulation environment and evaluated trajectory, the selected communication architecture has a substantial influence on modeled control-loop timing and consistency. Overall, the comparative analysis confirms that REST-only communication is unsuitable for real-time IoRT control loops due to excessive latency and variability, while MQTT-only communication lacks sufficient support for higher-level supervisory functions. The hybrid MQTT–REST architecture effectively integrates the strengths of both protocols, enabling reliable real-time execution alongside flexible system management. These findings support the hybrid communication strategy as the most consistent configuration among those evaluated under the present simulation conditions. Validation of its practical robustness and real-time capability requires repeated experiments using physical broker, cloud, edge-controller, and network hardware.

### Discussion and recommendations

The results confirm that the proposed IoRT-based robotic control framework achieves high kinematic accuracy while maintaining practical feasibility under cloud-assisted operation. Sub-millimeter reconstruction accuracy was consistently achieved, with RMS position and orientation errors of 0.066 mm and 0.206°, respectively. These results validate the precision of the analytical inverse kinematics formulation and demonstrate that cloud-based kinematic computation does not degrade motion accuracy when properly integrated into the control loop. Moreover, the symmetric distribution of errors across workspace points indicates predictable and stable numerical behavior rather than configuration-dependent instability. The multi-branch inverse-kinematics selection strategy further enhances system performance by evaluating the available discrete IK configurations at each target pose. By selecting the shortest joint-space path among all feasible solutions, total joint travel was limited to 7.404 rad across the complete trajectory, representing an estimated 54.3% reduction compared to random solution selection. This reduction supports smoother motion execution and may reduce unnecessary actuator movement and mechanical wear, demonstrating the value of systematic multi-branch IK selection within a cloud-assisted IoRT framework.

The communication protocol evaluation provides critical insights for IoRT system design. While RESTful communication offers simplicity and ease of integration with cloud services, the results clearly show that its performance limitations approximately 4.35 × higher average delay than MQTT, protocol overhead exceeding 70%, and vulnerability to timeouts render it unsuitable for real-time robotic control. In contrast, the hybrid MQTT–REST architecture consistently delivers low latency, high reliability, and superior temporal consistency. With an average delay of 0.210 s and a standard deviation of only 0.006 s, the hybrid approach outperforms both MQTT-only and REST-only configurations while retaining system-level manageability. The ability to propagate emergency commands within 50 ms further highlights its suitability for safety–critical industrial applications. Based on the comparative analysis, the hybrid MQTT–REST architecture is recommended as the default communication strategy for cloud-assisted IoRT robotic systems requiring a balance between real-time performance and system integration. MQTT-only communication remains appropriate for highly constrained networks where minimal overhead is essential, while REST-only communication is suitable for early-stage development and prototyping but should be avoided in production control loops. For cloud-integrated smart factory environments, the hybrid architecture provides the most effective solution by combining real-time responsiveness with scalable cloud-edge coordination. The principal discussion outcomes and their corresponding technical implications are concisely summarized in Table 11.

**Table 11:** Synthesis of Discussion Outcomes and Technical Implications.

Discussion aspect	Key observations	System-level implications
Kinematic accuracy	RMS position error of 0.066 mm and RMS orientation error of 0.206° under cloud-assisted execution	Analytical IK can be safely offloaded to the cloud without degrading motion precision
Error behavior	Symmetric and consistent error distribution across workspace	Predictable numerical stability independent of robot configuration
Multiple IK branches	A minimum of three valid analytical IK branches was available per trajectory point	Availability of multiple IK branches provides alternative feasible configurations under joint-limit and singularity-proximity constraints
Solution selection strategy	Multi-branch shortest-path IK selection reduced total joint travel by approximately 54.3% relative to random branch selection	Reduced unnecessary joint motion and improved joint-space continuity
Communication latency	REST-only communication incurred ~ 4.35 × higher delay and > 70% protocol overhead	REST unsuitable for real-time robotic control loops
Hybrid communication	Average delay of 0.210 s with σ = 0.006 s and no timeout events	Hybrid MQTT–REST architecture ensures low latency and high reliability
Emergency response	Emergency commands propagated within 50 ms using hybrid communication	Suitable for safety–critical industrial IoRT applications
Framework feasibility	Stable execution achieved under cloud-assisted control logic	Practical deployment possible within modern IoRT infrastructures

## Conclusion

This paper presented a cloud-assisted IoRT framework that integrates established analytical forward- and inverse-kinematics models within a distributed cloud–edge architecture for a six-degree-of-freedom robotic manipulator. The study does not claim a new analytical kinematic formulation for the PUMA 560; rather, it uses its classical closed-form kinematics as a non-iterative and interpretable computational basis for investigating cloud-assisted motion generation. The principal contribution is the architectural and operational integration of analytical kinematic computation, multiple-solution evaluation, communication, local validation, and edge-side execution within a unified simulation-based IoRT framework. In this architecture, inverse-kinematics computation and trajectory generation are assigned to the cloud, whereas joint-limit enforcement, safety checks, and command execution remain at the edge.

Simulation-based validation demonstrated that the proposed framework achieves high kinematic accuracy under cloud-assisted execution, with a root-mean-square position error of 0.066 mm and a root-mean-square orientation error of 0.206°. The observed symmetric and consistent error distribution across the workspace confirms the numerical robustness and configuration-independent stability of the analytical kinematic model. Furthermore, the multi-branch inverse-kinematics solution-selection strategy reduced total joint displacement by approximately 54.3% relative to random branch selection for the evaluated trajectory, contributing to smoother joint-space transitions and reduced unnecessary motion. Communication performance analysis revealed that hybrid cloud–edge interaction can maintain bounded latency and reliable command delivery, whereas REST-only communication introduces excessive delay unsuitable for real-time robotic control. Collectively, these results support the simulation-level feasibility of cloud-assisted, latency-aware analytical kinematic control while retaining command validation and time-sensitive execution functions at the edge. Dynamic closed-loop stability and torque-level control remain subjects for future experimental investigation.

### Future work

Future research will extend the proposed framework beyond kinematic modeling toward full dynamic-aware cloud-assisted robotic control. This includes incorporating dynamic models, torque-level control, and force–interaction constraints to support more complex manipulation tasks such as assembly, contact-rich operations, and human–robot collaboration. Experimental validation on physical robotic platforms will be conducted to assess real-world communication variability, sensor noise, and execution uncertainty under industrial operating conditions. In addition, adaptive and learning-based modules will be explored to enhance motion optimization, disturbance rejection, and task-level autonomy, while maintaining the interpretability and safety guarantees of analytical kinematic formulations. Future implementations will incorporate configurable communication-timeout thresholds and an edge-side fallback mechanism capable of maintaining a safe hold state or generating short-horizon trajectories locally when cloud communication becomes unavailable or exceeds the permitted latency bound. Finally, the proposed IoRT framework will be extended to multi-robot and collaborative scenarios, enabling coordinated cloud-assisted control of heterogeneous robotic systems within smart manufacturing environments.

## Data Availability

All data generated and analyzed during this study are included in this article.

## Declarations

### Software

MATLAB software was used as a platform for modeling the robotic arm and implementing the proposed algorithms.
